# Data-driven computational prediction and experimental realization of exotic perovskite-related polar magnets

**DOI:** 10.1038/s41535-020-00294-2

**Published:** 2020

**Authors:** Yifeng Han, Meixia Wu, Churen Gui, Chuanhui Zhu, Zhongxiong Sun, Mei-Huan Zhao, Aleksandra A. Savina, Artem M. Abakumov, Biao Wang, Feng Huang, LunHua He, Jie Chen, Qingzhen Huang, Mark Croft, Steven Ehrlich, Syed Khalid, Zheng Deng, Changqing Jin, Christoph P. Grams, Joachim Hemberger, Xueyun Wang, Jiawang Hong, Umut Adem, Meng Ye, Shuai Dong, Man-Rong Li

**Affiliations:** 1 Key Laboratory of Bioinorganic and Synthetic Chemistry of Ministry of Education, School of Chemistry, Sun Yat-Sen University, 510275 Guangzhou, China.; 2 School of Physics, Southeast University, 211189 Nanjing, China.; 3 Skolkovo Institute of Science and Technology, Bolshoy Boulevard 30, bld. 1, Moscow 121205, Russia.; 4 State Key Laboratory of Optoelectronic Materials and Technologies, School of Materials, Sun Yat-Sen University, 510275 Guangzhou, China.; 5 Beijing National Laboratory for Condensed Matter Physics, Institute of Physics, Chinese Academy of Sciences, 100190 Beijing, China.; 6 Songshan Lake Materials Laboratory, 523808 Dongguan, Guangdong, China.; 7 Institute of High Energy Physics, Chinese Academy of Sciences, 100049 Beijing, China.; 8 Spallation Neutron Source Science Center, 523803 Dongguan, China.; 9 NIST Center for Neutron Research, National Institute of Standards and Technology, Gaithersburg, MD 20899-6102, USA.; 10 Department of Physics, Rutgers, The State University of New Jersey, Piscataway, NJ 08854, USA.; 11 NSLS-II, Brookhaven National Laboratory, Upton, NY, USA.; 12 Institute of Physics, School of Physics, University of Chinese Academy of Sciences, Chinese Academy of Sciences, P. O. Box 603, 100190 Beijing, China.; 13 II Physikalisches Institut, Universität zu Köln, 50937 Köln, Germany.; 14 School of Aerospace Engineering, Beijing Institute of Technology, 100081 Beijing, China.; 15 Department of Materials Science and Engineering, İzmir Institute of Technology, Urla, 35430 İzmir, Turkey.; 16 State Key Laboratory of Low Dimensional Quantum Physics and Department of Physics, Tsinghua University, 100084 Beijing, China.; 17 These authors contributed equally: Yifeng Han, Meixia Wu.

## Abstract

Rational design of technologically important exotic perovskites is hampered by the insufficient geometrical descriptors and costly and extremely high-pressure synthesis, while the big-data driven compositional identification and precise prediction entangles full understanding of the possible polymorphs and complicated multidimensional calculations of the chemical and thermodynamic parameter space. Here we present a rapid systematic data-mining-driven approach to design exotic perovskites in a high-throughput and discovery speed of the *A*_2_*BB*’O_6_ family as exemplified in *A*_3_TeO_6_. The magnetoelectric polar magnet Co_3_TeO_6_, which is theoretically recognized and experimentally realized at 5 GPa from the six possible polymorphs, undergoes two magnetic transitions at 24 and 58 K and exhibits helical spin structure accompanied by magnetoelastic and magnetoelectric coupling. We expect the applied approach will accelerate the systematic and rapid discovery of new exotic perovskites in a high-throughput manner and can be extended to arbitrary applications in other families.

## INTRODUCTION

Conventional discoveries of new materials by trial and error require many costly experiments. However, recent rapid development of data accumulation and computational power has enabled rapid materials innovation via synergetic integration of experiment, computation, and theory^1–4^. The emerging data-driven prediction and experimental realization have been extensively applied to solid-state materials with designed combinations of crystal structures and technical applications^5,^ such as battery materials^6^, superhard materials^7^, transparent conducting oxides^8,9^, and photovoltaic materials^10^. One rational design approach is to determine the ground state, or relevant property of interest, for possible structure archetypes with a user-confined threshold, followed by a composition screening for optimized targets^11,12^. This procedure accumulatively guides prospective inverse-design^13^, especially for the superior metastable materials that can only be prepared under stringent and costly synthesis, such as in the high temperature and pressure (HPT) exploration of corundum-derived polar magnets^14.^

Double-corundum-related polar magnets have been extensively studied for multiferroic and magnetoelectric spintronics. Their crystal structures feature small cations at *A*-sites and rock-salt ordering of *B*O_6_–*B*’O_6_ related to exotic *A*_2_*BB*’O_6_ perovskites^14–16^. In principle, they can be assembled to form numerous polar magnets in *A*_2_*BB*’O_6_ through compositional flexibility. However, only <20 such compounds are reported to date, mostly limited by the costly and stringent HPT synthesis conditions. The conventional geometric descriptors, such as the Goldschmidt tolerance factor (*t*)^17^, one-dimensional tolerance factor (*τ*)^18^, octahedral factor (*μ*), and atomic packing fraction (*η*)^19,20^, can predict stable structures with an accuracy >90% in the conventional perovskites with large *A*-site cations. However, in exotic perovskite-related phases with small *A*-site cations, the geometry-based prediction is insufficient to discriminate between the competing polymorphs. It is therefore beneficial to use data-mining to learn from the past and predict the future research on this theme. Thermodynamics has been proven to play a central role in the phase stability of competing polymorphs in exotic perovskite-related materials, for example, by means of HPT synthesis in *AB*O_3_. The ilmenite *R*-3 phases *A*TiO_3_ (*A* = Mn^21,22^, Fe^23^, Zn^24^) and MnVO_3_ can be transformed to LiNbO_3_ (LN, *R*3*c*) and perovskite (*Pnma*) type structure^25,26^, respectively; the bixbyite (*Ia*-3) type ScFeO_3_ undergoes conversion into LN and perovskite phase in sequence at increased pressure^27,28.^ Therefore, prediction of the experimental conditions for the desired polymorph (if they exist) is the key to avoid or minimize costly trial and error.

**Fig. 1:**
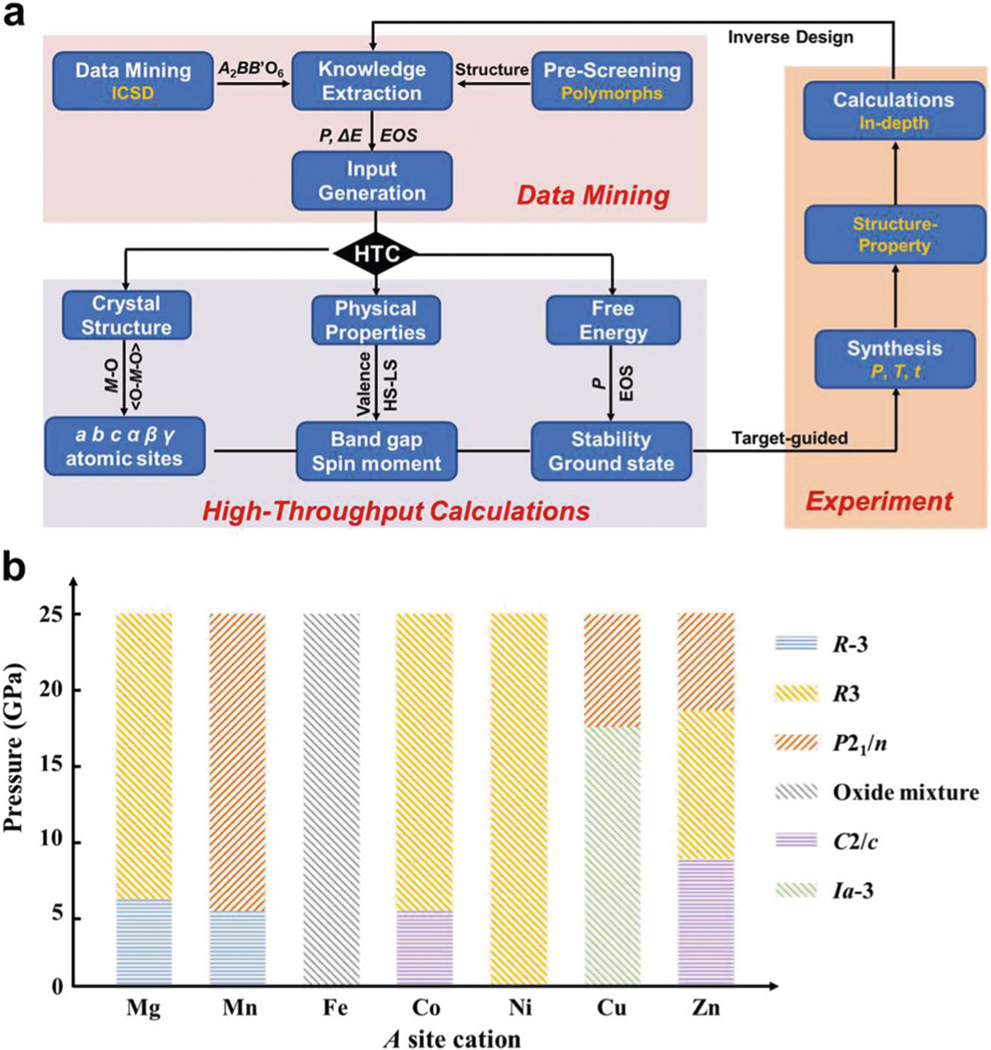
Discovery of exotic perovskite-related materials via data-driven approach. **a** The workflow of function-oriented searching (composition screening) and designing of new exotic *A*_2_*BB*’O_6_ materials via seamless integration of data mining, high-throughput computing (HTC), and experiment. (1) Data mining: the synthesis conditions and properties of existing polymorphs feed the starting models and input generation for DFT calculations—the starting models and correlated pressure (*P*) effect, Δ*E* (equivalent to Δ*H* in first-principles calculations), and equation of state (EOS); (2) HTC: the proposed crystal structure (including unit cell parameters, atomic positions, metal–oxygen bond length (*M*–O) and angle (<O–*M*–O>)), corresponding physical properties (band gap and spin moment in light of the valence state, electron configuration of high or low spin (HS–LS) states), and synthesis conditions (*P*-dependent energy evolution) enable target-guided synthesis; (3) Experiment: precise synthesis for desired polymorph according to predicted conditions allows in-depth structure–properties and calculations for further inverse design. **b** Predicted pressure-dependent polymorph evolution of *A*_3_TeO_6_ (*A* = Mg^2+^, Mn^2+^, Fe^2+^, Co^2+^, Ni^2+^, Cu^2+^, Zn^2+^) up to 25 GPa, in which Fe_3_TeO_6_ does not form a single phase and only an oxide mixture can be obtained.

The formation of possible polymorphs in exotic perovskite-related phases may vary under applied thermodynamic conditions (temperature, pressure, etc.), where pressure is an easier thermodynamic parameter for preliminary prediction regardless of the temperature effect^29^. Recently, experiment-based first-principles density functional theory (DFT) calculations in light of the Murnaghan equation of state (EOS) for solid^30^ explicitly pinpointed that the phase stability between different polymorphs can be well evaluated by the phase diagram of the relative enthalpy (Δ*H*) and pressure (*P*)^24,31–33^. For example, the pressure-dependent Δ*H* evolution of the *Pnna*, *LN* (*R*3*c*), ilmenite (*R*-3), and perovskite (*Pnma*) polymorphs well explained the *Pnn*2-to*-LN* transition in LiSbO_3_ around 8 GPa^31^. These findings advanced a paradigm for computationally assisted identification of new materials by pressure-dependent Δ*H* calculations over all possible polymorph candidates. Data-mining of the Inorganic Crystal Structure Database and literature screening dug out only 68 known compounds (Table S1 in Supporting Information (SI), 65 prepared and 3 calculated) of the predicted 13,000 plus possible compounds in exotic *A*_2_*BB*’O_6_, leaving a huge opportunity for new materials exploration. Crystal structures of the reported 68 compounds can be classified into six different polymorphs as shown in Fig. S1, which enables accelerated, function-oriented discoveries of new materials in a high-throughput computing (HTC) manner. However, it is a challenge to prioritize selection of synthetic targets in exotic *A*_2_*BB*’O_6_ following the workflow in Fig. 1a. Here we selected the *A*_3_TeO_6_ system to illustrate the predictive capacity, because (1) it has the large structural diversity (5 polymorphs reported except LiSbO_3_-derivative *Pnn*2 as highlighted in Table S1) and affords a representative platform. And (2) its multifunctional physical properties have been extensively explored in the known materials to test the scope of our approach. And (3) the energy shift from different proposed ferromagnetic/antiferromagnetic (FM/AFM) structures does not dominate the ground states of the competing polymorphs^34^ and can be ignored to reduce the dimensionality of the calculations for a reliable and timely exploration. Previous calculations have precisely predicted the pressure-induced polymorph manipulation in Mn_3_TeO_6_, which has been confirmed by experiment. Although the high-pressure (HP) *P*2_1_/*n*-polymorph exhibits a magnetodielectric effect and about 13 K higher AFM ordering temperature than the ambient-pressure (AP) *R*-3 phase in Mn_3_TeO_6_, energetically the expected polar and magnetic *R*3-type structure is not favored and thus lacks any multiferroic response^34^. In this work, we performed target-guided research following Fig. 1a, predicted the pressure-dependent polymorph evolution of *A*_3_TeO_6_ (Fig. 1b, where *A* is possible divalent cations with ionic radius no larger than that of Mn^2+^), and paradigmatically singled out the Δ*H*–*P* phase diagram upon the six possible polymorphs (Fig. S1) of Co_3_TeO_6_ (CTO), experimentally realized the HPT synthesis of the predicted polar magnet, and intensively characterized its crystal and magnetic structures and physical properties.

## RESULTS

**Fig. 2:**
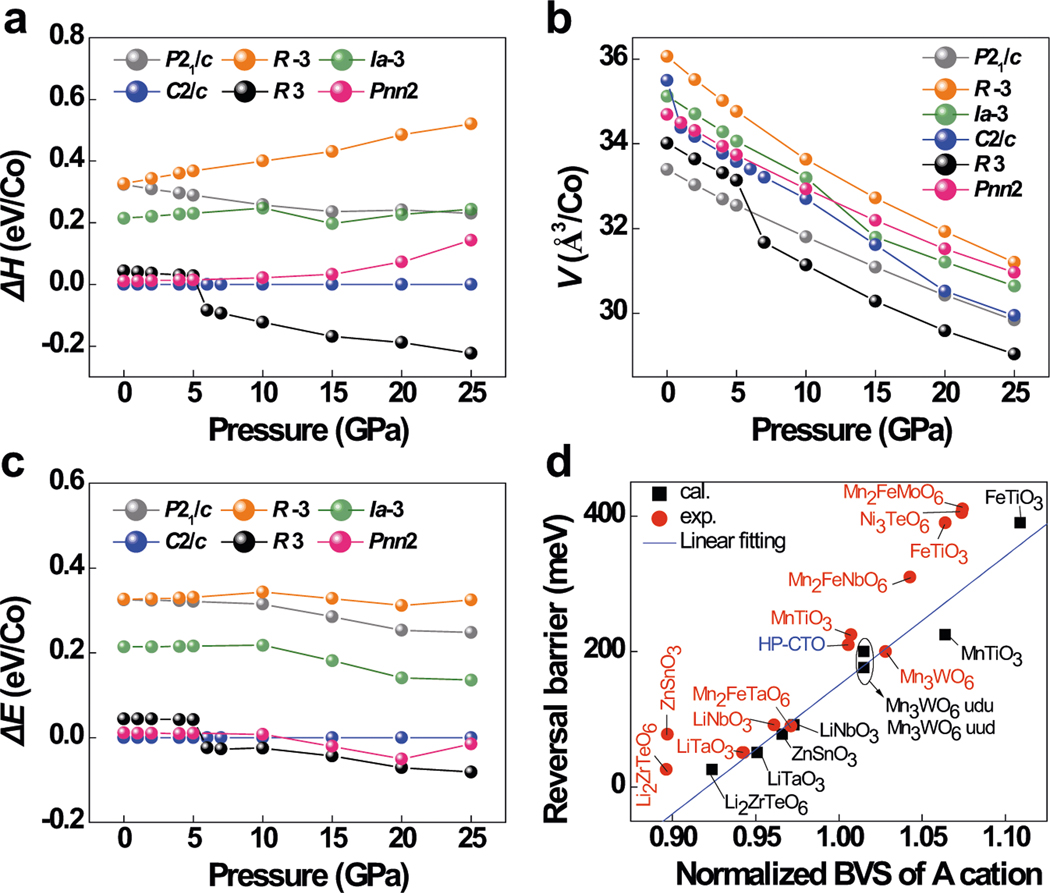
Theoretical calculation results on CTO. Calculated pressure dependencies of the **a** relative enthalpies, **b** volume, and **c** relative total energy of CTO in the six possible polymorphs. The *Y*-axis demonstrates the relative enthalpies, volume, and relative total energy divided by the number of Co in a unit cell for better comparison. **d** Scatterplot of domain-wall-mediated reversal barrier versus normalized BVS (divided by the valence charge) of the *A*-cations, where the black squares denote the normalized BVS calculated (cal.) from the optimized crystal structure, while the red spheres are for the normalized BVS from the experimental (exp.) CIFs (crystallographic information files). The “u” and “d” in Mn_3_WO_6_ represent for the “up” and “down” spin arrangement from ref. ^36^, respectively.

Prediction of the phase stability and physical properties of CTO Structural data-driven HTC on *A*_3_TeO_6_ system following the workflow in Fig. 1 suggest that CTO undergoes a pressure-induced centrosymmetric to polar structure transition as summarized in Fig. 2a–c. The AP-prepared Co_3_TeO_6_ (monoclinic *C*2/*c*, denoted as AP-CTO) crystallizes in *C*2/c symmetry (Fig. S1c) and demonstrates type-II multiferroicity with complicated incommensurate magnetic ordering below 26 K^27,35^. The evolution of pressure-dependent Δ*H* (or relative free energy (Δ*E*) per cobalt atom) in a unit cell of CTO calculated on the six possible polymorphs is plotted in Fig. 2a, where the Δ*H* variation of AP-CTO (*C*2/*c* phase) is set as the baseline with increasing pressure for comparison. In the pressure range below 5 GPa, the *C*2/*c*-type phase is the most stable polymorph in terms of enthalpy by comparison with the energetically comparable *Pnn*2 and *R*3 counterparts, giving initial Δ*H* difference of 0.04 eV/Co between *R*3 and *C*2/*c*. The relative enthalpy of the *R*3 phase gradually decreases with increasing pressure and experiences an abrupt drop from 0.03 to −0.08 eV/Co around 5 GPa, accompanied by a volume reduction from 33.14 to 31.67 Å^3^/Co (Fig. 2b) and Δ*E* decrease from 0.042 to −0.024 eV/Co (Fig. 2c). The above results indicated that the *R*3 phase is energetically more stable than the others above 5 GPa, and a phase transition from *C*2/*c* structure to polar *R*3 phase is thus expected. There is no further phase transition up to 25 GPa as reflected by Fig. 2a–c. Unlike Mn_3_TeO_6_ and other exotic perovskites, the higher-pressure *P*2_1_/*n* structure is energetically unfavorable in CTO. The predicted polar *R*3 structure inspired further estimation of spontaneous polarization (*P*_S_) in this HP Co_3_TeO_6_ (HP-CTO), which is calculated to be around 75.3 μC/cm^2^ from the optimized crystal structure based on the point charge model. The polarization switching barrier in polar corundum derivatives can be evaluated by the local bonding environment of the mobile *A* cations as measured by normalized bond valence sums (BVS divided by valence charge)^36,37^. As shown in Fig. 2d, the domain-wall-mediated reversal barrier of HP-CTO (~180 meV) estimated from the optimized crystal structure is comparable to that of the multiferroic Mn_3_WO_6_ analog^38^ but smaller than that of Ni_3_TeO_6_ (~400 meV)^39^. Therefore, experimental realization of the predicted *R*3-type HP-CTO will afford a promising multiferroic polar magnet without any lone-pair electron or second-order Jahn–Teller distorted *d*^0^ ions.

### Crystal structure and phase stability of HP-CTO

The pressure-dependent polymorph manipulation of CTO experimentally coincides well with theoretical calculations. AP-CTO (monoclinic, *C*2/*c*) indeed transforms into HP-CTO (hexagonal *R*3) at 5 GPa and 1023 K (Fig. S2). The crystal structure of HP-CTO, decently refined in the Ni_3_TeO_6_ model from synchrotron and neutron powder diffraction (SPXD and NPD, Fig. S3 and inset) data, is listed in Table S2. The selected interatomic distances, bond angles, and observed BVS calculations are listed in Table S2. There is no structural phase transition observed down to 5 K from NPD. Structural parameters obtained from the low-temperature NPD (5 and 45 K) data are in Tables S3–S9. The Co–O bond length varies between 2.013(6) and 2.22(1) Å and averages to <Co-O> of 2.12 Å in HP-CTO, which is nearly identical to the <Co-O> distance of 2.13 Å in CoO_6_ of AP-CTO. The average <Te-O> bond length (1.926 Å) in HP-CTO is very comparable with those in *A*_3_TeO_6_ (*A* = Co, Mn, Ca, Cd, Cu, and Mg) ranging between 1.908 and 1.969 Å. Both the structural analysis and X-ray absorption near-edge spectroscopy (XANES) (Fig. S4) data clearly manifested Co^2+^ (and by inference Te^6+^) in HP-CTO. The large structural distortion results in calculated *P*_S_ of 81.3 μC/cm^2^, close to the value (75.3 μC/cm^2^) estimated from the optimized structure. The observed lattice volume of HP-CTO (35.8 Å/Co) is ~4.27% smaller than that of AP-CTO (37.4 Å/Co), in reasonable agreement with the prediction in Fig. 2. Understandably, HP synthesis favors the polymorph with smaller lattice volume, which is, however, metastable and not the ground state at AP. Figure S5 presents the room-temperature laboratory powder X-ray diffraction (XRD) patterns of the HP-CTO after annealing at variable temperatures at AP, which shows that the HP-CTO phase can persist up to 1123 K before irreversibly converting back to the AP-CTO above 1143 K. Kinetically, the stable temperature region of HP-CTO is wide enough for practical applications.

### Magnetic properties of HP-CTO

**Fig. 3:**
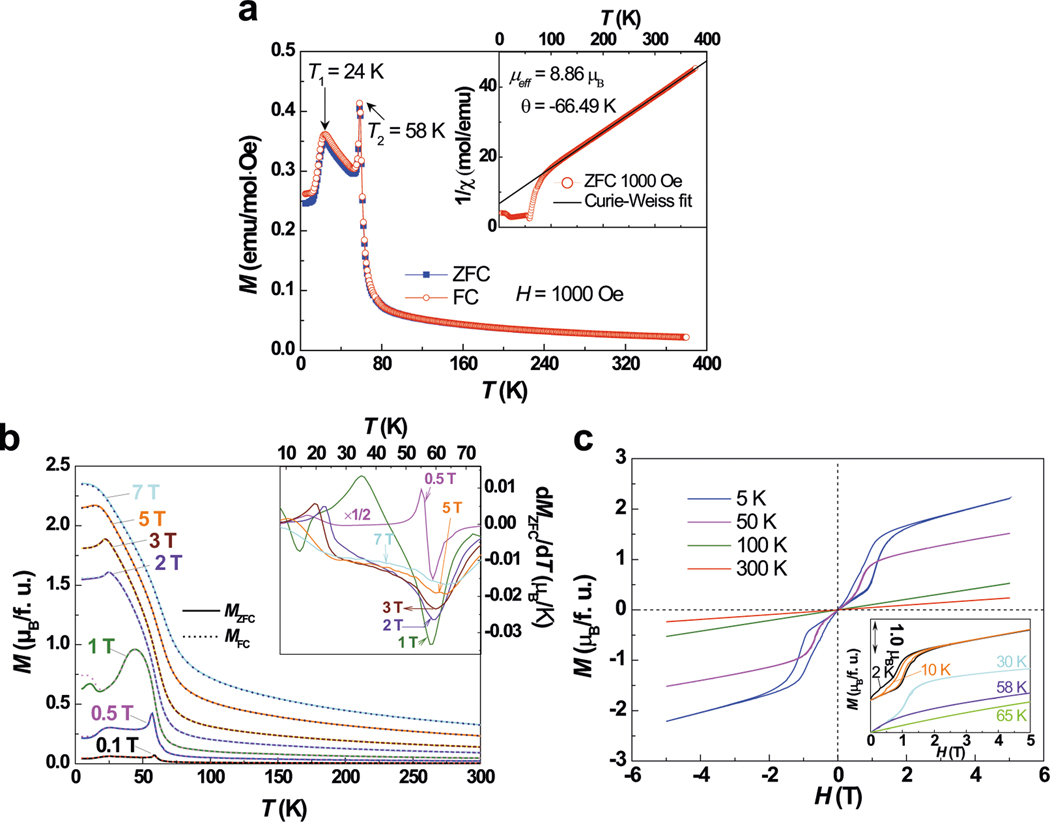
Magnetic properties of HP-CTO. **a** Thermal evolution of the ZFC and FC mode magnetization (*M*) and the inverse susceptibility (*χ*^−1^) (inset) of HP-CTO collected with a field of *H* = 0.1 T. **b** *M*(*T*) curve at a series of magnetic fields between 0.1 and 7 T. Inset shows the numerical d*M*/d*T* derivatives for the ZFC-*M*(*T*) curves in the main part of the figure. The *H* = 0.5 T curve is reduced by the factor 1/2 for clarity. **c** Isothermal magnetization curves measured at 5, 50, 100, and 300 K between −5 and 5 T. Inset shows the *M*–*H* curves measured at 2, 10, 30, 58, and 65 K between 0 and 5 T.

The temperature-dependence magnetization (*M*) measured at a field of *H* = 1000 Oe is displayed in Fig. 3a. The onsets of two obvious magnetic transitions at *T*_1_ ~ 24 K and *T*_2_ ~ 58 K are higher than the transition temperatures in AP-CTO (Supplementary Fig. S6). At temperature above ~130 K, HP-CTO follows the Curie–Weiss law *χ* = *C*/(*T* − *θ*). The negative *θ* (−65.4 K) suggests predominantly AFM interactions between the Co ions. With decreasing temperature between 80 and 60 K, precursive to the transition, there is a rapid rise of *M* and a concomitant downward curvature of the *χ*^−1^. The authors attribute this to the transverse canting response of the AFM fluctuations to the field and will be discussed further below. The effective magnetic moment derived from the Curie–Weiss fit of 1/*χ*(*T*) over the paramagnetic region (inset of Fig. 3a) is *μ*_eff_ = 5.11 *μ*_B_/Co^2+^. This value is larger than the theoretical effective magnetic moment of spin-only contribution (3.87 *μ*_B_/Co^2+^) and is also larger than the AP-CTO (with *μ*_eff_ of 4.74 *μ*_*B*_/Co^2+^). However, this derived *μ*_eff_ is close to the theoretical value of 6.02 *μ*_B_/Co^2+^ with an orbital moment *L* = 3, suggesting that the Co^2+^ orbital moment is not fully quenched in HP-CTO^40^.

Figure 3b shows the *M* versus *T* curves collected in the zero-field cooling (ZFC) and field-cooling (FC) modes at magnetic field between 0.1 and 7 T. The onset of magnetic order with an AFM component is associated with the inflection point on the low temperature side of the *M*(*T*) peak. At 1 T, the AFM transition at *T*_2_ is weakened and shifted to lower temperature. With increasing magnetic field, this AFM order is substantially modified. On the other hand, the *T*_1_ AFM transition gradually disappears upon increasing magnetic field. This behavior is similar to that of Mn_2_FeWO_6_ and Mn_2_MnWO_6_ and demonstrates the complex magnetic structure of HP-CTO and possible multiferroicity driven by the coupling between spin, charge, and orbital degrees of freedom^15,38^. Inset of Fig. 3b shows the numerical d*M*/d*T* derivatives for the ZFC curves. The *H* = 0.5 T curve is reduced by the factor 1/2 for clarity. The prominent broad negative peaks in the d*M*/d*T* curves are consistent with a non-linear canted moment field response associated with transverse AFM fluctuations/correlations. As shown in Fig. 3c, the *M*–*H* curve shows a step-like character below 50 K, supporting the presence of a first-order (spin-flop) field-induced phase transition.

### Magnetic structures of HP-CTO

**Fig. 4:**
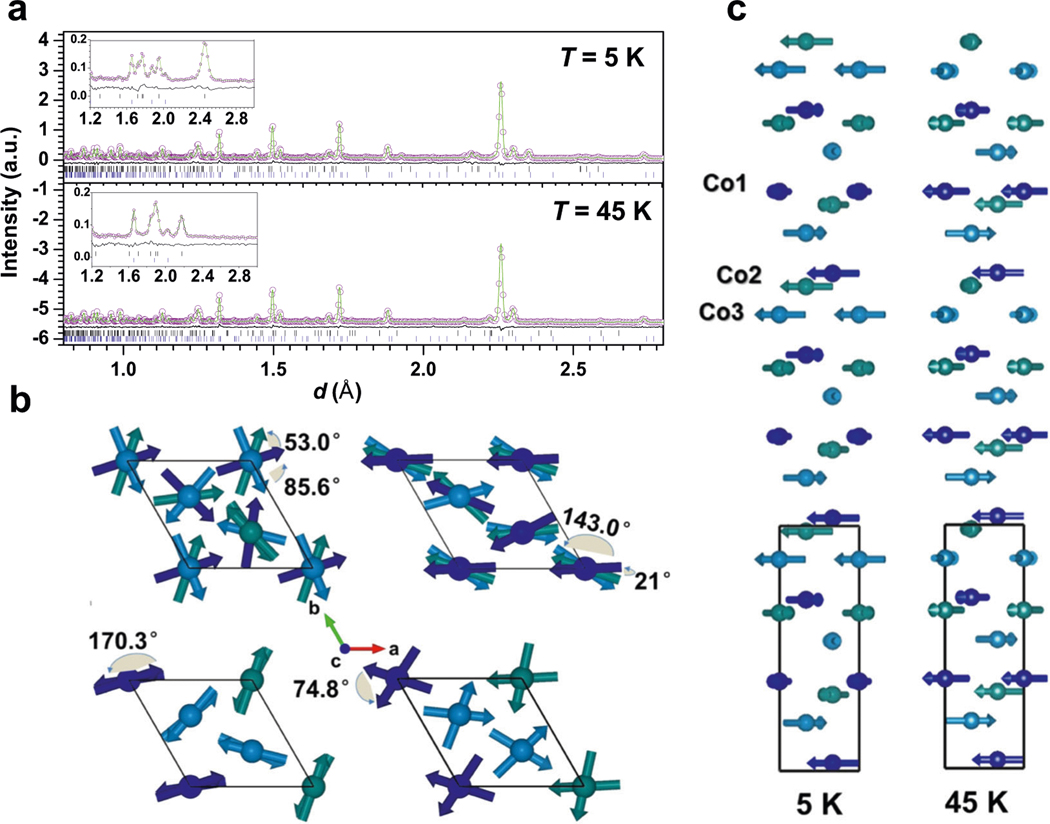
Magnetic structure of HP-CTO determined from in situ variable temperature powder neutron diffraction measurements. **a** Experimental, calculated, and difference patterns from refinements of the NPD data at *T* = 5 and 45 K (high-resolution bank 2 (58°, ~1–9.6 Å)), insets show the refined results of bank 3 (90°, ~0.7–6.6 Å) data. The blue and black bars mark the reflection positions for the nuclear reflections and magnetic satellites, respectively. **b** The arrangement of magnetic moments at *T* = 5 and 45 K. The unit cell for the nuclear structure is outlined. The arrangement of magnetic moments at *T* = 5 K (left panel of **c**) and *T* = 45 K (right panel of **c**) viewed along [001]. The blue spheres mark the Co1 atoms, green—Co2, light blue—Co3.

NPD data at low temperatures were recorded to better depict the complex magnetic behavior of HP-CTO. All magnetic reflections can be possibly indexed as satellites of the allowed nuclear reflections, using a wavevector incommensurate with the lattice and oriented along the c* axis, since the magnetic peak shift converges with temperature increase between 5 and 50 K as shown in Fig. S7. Figure 4a shows the magnetic reflections at *T* = 45 and 5 K, respectively. The *T* = 45 K pattern is indexed with a propagation vector (Tables S4 and S5) *k* = [0, 0, *γ*] (*γ* = 0.2072(12)), while the *T* = 5 K pattern with *γ* = 0.5252(8). HP-CTO adopts a constant moment spiral magnetic structure consisting of AFM helixes propagating along the с axis (the helixes are associated with the Co1, Co2, and Co3 atomic chains; Fig. 4b). At 5 K, the magnetic moments of the Co1 chain is rotated about ≈53.0° with respect to that of Co2 and about ≈85.6° with respect to Co3 (Fig. 4c, left). However, at *T* = 45 K the rotation angles are different: ≈21.0° between the Co1 and Co2 moments and ≈143.0° between the Co1 and Co3 moments. (Fig. 4c, right). The magnetic moment of each subsequent atom along the chain rotates relative to the previous one by ≈170.3° and 74.8° at *T* = 5 and 45 K, respectively. The ordered magnetic moment of the Co atoms is constant being equal to 2.97(1) *μ*_B_ at *T* = 5 K and 2.25(1) *μ*_B_ at *T* = 45 K.

The structural evolution of HP-CTO between *T* = 5 and 90 K was determined by in situ variable temperature NPD studies (Fig. S8 and Table S6–10). Thermal-expansion response was observed below the first magnetic transition region *T*_1_ (~24 K) upon heating, where *a* and *c* reached the first anomaly with a minimum of 5.1984(8) Å and maximum of 13.842(4) Å, respectively, and then follow a sharp negative (−0.006 Å) thermal expansion (NTE) along *c* to 13.836(4) Å and a steeper positive (0.001 Å) thermal expansion (PTE) to 5.1994(6) Å till the second magnetic transition *T*_2_ (~58 K) region (Fig. S9a). In contrast, *c* and *a* behaved inversely after the second anomaly, and the PTE of *c* and NTE of *a* are not so robust compared to those following the first transition. These anomalies around the magnetic transition temperatures suggest the presence of strong magnetostriction coupling in HP-CTO, which yield asymmetric “W”-shape volumetric change between *T*_1_ and *T*_2_ (Fig. S9b).

### Magnetoelectric properties of HP-CTO

**Fig. 5:**
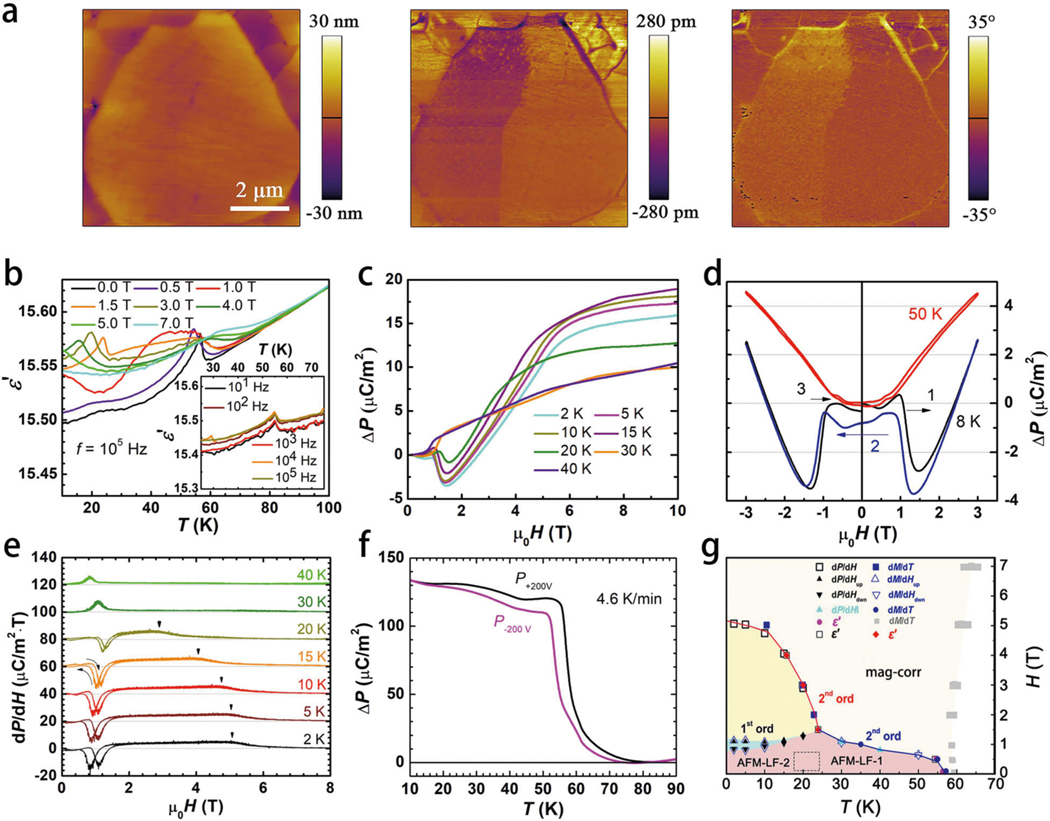
The polar-nature-related physical properties of HP-CTO. **a** Piezoelectric performances: the topography (left), amplitude signal (middle), and phase image (right) of the out-of-plane piezo response. **b** Temperature dependence of dielectric constant at various magnetic fields. Inset shows the dielectric constant at different frequencies. **c** Temperature dependence of electric polarization. **d, e** Magnetic field dependence of the electric polarization at various temperatures. For **c**, the data were obtained upon sweeping the magnetic field up. **f** The d*P*/d*H*–*μ*_0_*H* plots derived from **c**, which has a non-hysteretic peak at ≥30 K, indicating a second-order phase transition. At lower *T*, we see two features: a minimum that clearly shows hysteresis and a step that shifts toward higher fields at low temperatures. **g** A schematic low temperature *H*–*T* magnetic phase diagram based on the *M*–*H*, *P*–*H*, *M*–*T*, and *ε*–*T* measurements. The detailed discussion and justification of the electrical and magnetic measurements and indicators of the transitions in the phase diagram are discussed at greater length in SI.

The computation-assisted prediction and precise synthesis of HP-CTO enable further insights into the polar-nature-based properties such as piezoelectric, ferroelectric, and magnetoelectric behaviors. Figure 5a present the topography and piezo response force microscopy (PFM) phase and amplitude images for the polished polycrystalline HP-CTO at room temperature, where different polarization states correspond to the bright and dark contrasts in the phase and amplitude images (Fig. 5a middle and right), which are independent from the topography (Fig. 5a, left). The observation of ferroelectric domain inside a hexagonal grain indicates the electromechanical coupling of HP-CTO at room temperature, originating from the non-centrosymmetric structure of this new material. Temperature dependence of the dielectric constant *ε*′ of HP-CTO at various magnetic fields is shown in Fig. 5b, inset of which shows the temperature-dependent dielectric constant at frequencies from 10 Hz to 100 kHz. A peak in the dielectric constant appears at ~58 K, which coincides with the magnetic transition temperature (*T*_2_ ~ 58 K). The position of this peak does not change with increasing frequency, indicating the intrinsic nature of the dielectric anomaly. As shown in Fig. 5b, in magnetic fields <1 T, the dielectric peak at ~58 K remains intact with a slight downshift to low temperatures. However, above 1 T, the peak at ~58 K becomes broad and gradually inconspicuous with further increase of the magnetic field. On the other hand, above 1 T, another dielectric peak appears at about 24 K. This peak is also magnetic field dependent and shifts to lower temperatures with increasing magnetic field. The temperature of this second dielectric anomaly coincides with the magnetic transition temperature *T*_1_ ~ 24 K. The above results indicate that the two different magnetic states observed in the magnetic properties are reflected in the dielectric constant by two distinct dielectric anomalies.

Magnetic order also induces changes in the electrical polarization and the magnetic order induced polarization is magnetic field dependent. Hence HP-CTO is magnetoelectric. The temperature dependence of electric polarization in HP-CTO is displayed in Fig. 5c. Polarization develops below the onset of AFM order at *T*_2_. However, the electric polarization in HP-CTO cannot be switched as the direction of the applied poling electric field is reversed. Similar to isostructural Ni_3_TeO_6_^41^, the nature of HP-CTO is therefore pyroelectric, rather than ferroelectric. The observed polarization in HP-CTO is smaller by a factor of approximately 20 compared to Ni_3_TeO_6_, which is probably caused by the polycrystalline nature of the HP-CTO sample.

The isothermal changes of the electric polarization by magnetic field sweeping at various temperatures are displayed in Fig. 5d, f. HP-CTO shows magnetoelectric coupling for all measured temperatures. Above 2 T, the polarization increases abruptly, while tending to saturate at a field of 7 T. The polarization measured at 8 and 50 K was chosen to analyze the magnetic field dependence in detail. As shown in Fig. 5e, the polarization was recorded by sweeping the applied fields between −3 and 3 T. The polarization change at 8 and 50 K are quite different. At 8 K, *P* decreases dramatically above ~1 T with increasing *H* and reaches ~2.7 μC/m^2^ in magnitude around 1.5 T. While decreasing *H* from 3 T, *P* obviously exhibits hysteresis and does not recover the initial value at *H* = 0 T, possibly due to the creation of multiple magnetoelectric domains with opposite *P*. However, at 50 K, polarization increase shows quadratic behavior, and very limited hysteresis is observed. A schematic low temperature–magnetic phase diagram for HP-CTO, based on the *M*–*T*, *M*–*H*, *P*–*H*, and *ε*–*T* curves, is plotted in Fig. 5g. The presence of FM/ferrimagnetic correlations in HP-CTO in finite *H* fields was noted in the *M*, d*M*/d*T*, and *M*(*H*) results in Fig. 3. In particular, the negative peak in d*M*/d*T* curves in Fig. 3b (inset), starting near 58 K for *H* = 0 and systematically moving to higher temperature while broadening with increasing *H*, is characteristic of a canted (correlations/fluctuations) moment magnetic field response. Accordingly, the central temperature and width of these negative peaks in d*M*/d*T* curves are indicated in Fig. 5g by gray rectangles. The solid square data points represent the inflection point on the low temperature side of the *M*(*T*) curves (positive d*M*/d*T* maximum) in Fig. 3b. The solid line for view represents the locus of a second-order magnetic transition associated with the development of an AFM-type order parameter. At temperatures <20 K, the field-induced change is clearly a hysteretic first-order (spin-flop) phase transition. The increasing/decreasing field hysteresis is below the limit of the experimental certainty above 30 K. In the same temperature range, the field-induced transition empirically merges with the second-order temperature-induced transition (blue solid line). Accordingly, it is assumed that the temperature and field-induced transitions, respectively, represent the *T* and *H* crossing of the same second-order transition. Detail of phase diagram delineation can be found in Figs. S10 and S11. Although the BVS-based domain-wall-mediated ferroelectric reversal barrier of HP-CTO is estimated to be comparable with that of the magnetoelectric Mn_3_WO_6_ as shown in Fig. 2d, experimentally HP-CTO does not exhibit any expected displacive ferroelectric response as in Mn_3_WO_6_^38^. Absence of ferroelectricity in HP-CTO might be attributed to the extrinsic defects and grain boundary effect since the *P*–*E* loop measurements were conducted in polycrystalline sample, or the intermediate state of possible ferroelectric switching renders a metallic state, and defeats any polarization reversal as found in Mn_2_FeWO_6_^15^. Single domain crystal or thin-film samples of HP-CTO would be valuable for further investigation. According to spin current or inverse Dzyaloshinskii–Moriya (DM) interaction, there is no macroscopic polarization generated in this kind of non-collinear and screw magnetic structure with components of the Co^2+^ moments in the *ab* plane^42,43^. As shown in Fig. S9, the lattice parameters change abruptly below *T*_2_ = 58 K. This lattice variation is thought to be due to magnetostriction below *T*_2_, which is also the origin of magnetoelectric properties of HP-CTO^38,44^.

## DISCUSSION

The ultimate goal of computationally assisted identification of new materials is to predict the stable (metastable) compounds at various conditions with the combination of certain crystal structure and desired properties, especially for those difficult to reach experimentally. Our big data-driven high-throughput computation enables quick predictions of the stable crystal structure, providing efficient search for materials with optimal properties. The first-principles DFT calculations in light of EOS for solid can effectively advance and accelerate the discovery of exotic perovskites in *A*_2_*BB*’O_6_ with small *A*-site cations, in that the pressure-dependent evolution of Δ*H* and Δ*E* can predict the thermodynamically stable pressure region for the six categorized polymorphs up to 25 GPa—the pressure limitation of large volume press. A new polar magnet, HP-CTO, was successfully isolated from the *A*_3_TeO_6_ family and experimentally prepared around 5 GPa. HP-CTO emerges as a new *d*^0^ or lone-pair electron ion-free polar perovskite with magnetostriction and magnetoelectric coupling. This work provides a model for illustrating how big data-driven approaches can be applied in the prediction of exotic perovskite. The pressure-dependent Δ*H* determination with compositional screening of the possible polymorphs extracted from known databases alternatively guides the rational design of exotic perovskites better than the alternative geometry-based descriptors, such as the conventional and newly developed tolerance factors and octahedral factor^18,19^.

Our results show relative accuracy of generating the Δ*H*–*P* phase diagram of each polymorph without a priori knowledge of the magnetic structure. The spin structures are unknown before input generation and generally restricted collinear magnetic structure models are applied in first-principles DFT calculations. Moreover, it is not possible to set the real spin structure for calculations when incommensurate modulation arises as in Mn_3_WO_6_ and HP-CTO (Fig. 3). The consistently successful prediction and experimental realization in *A*_3_TeO_6_ indicated that the energy resolution (μeV) of magnetic DM interactions does not affect the overall trend of predicted results^34,45^, and thus the simplest collinear FM ordering can be set for calculations. One should note that the calculated density of states and band gap could be much less accurate using the simplest FM model, and a more appropriate scenario would need to be adopted. For example, the calculated band gap of HP-CTO is around 0.1 eV using the simplest FM model, which is, however, far from the observed case, since experimentally HP-CTO is very insulating and its resistivity is beyond the measurement range of our instruments. The proposed collinear magnetic structure also rules out possible type-II multiferroics in centrosymmetric crystal structure, which need to be conversely validated by experiments. In addition, we have not conducted any precise prediction of the system with strong spin–orbit coupling, for instance, in *A*_2_*BB*’O_6_ with 4*d* and 5*d* transition metals. The estimated energetic contribution of spin–orbit interactions, which can be several orders of magnitude higher than that of magnetic DM interactions, requires multidimensional calculations and is by no means an easy job. A feasible approach is to perform confirmatory calculations on the very limited known compounds for inverse design and knowledge accumulation before any reliable and supervised high-throughput computation.

Thermodynamics plays a central role in the prediction of synthesizable metastable polymorphs. Following Fig. 1, we can precisely predict the synthesis pressure of the desired polymorph for CTO according to the phase-stability diagram proposed at 0 K. However, the synthesis temperature remains unknown and mostly relies on chemical intuition and synthesis experiences. For example, HP-CTO was found to be synthesizable between 1073 and 1573 K; it was either not formed or melted if the temperature is <1073 K or >1573 K at 5 GPa. Therefore, combined predictions with other approaches, such as Monte Carlo simulation, particle swamp optimization, and/or phase-field simulation, are needed to figure out the synthesis temperature and achieve more precise predictions and cost-effective experiment. We expect the procedure can be tailored to a wider variety of materials besides exotic perovskites and enable large-scale screening of new materials in the near future.

## METHODS

### Computational descriptions

Calculations on relaxed structure were carried out at various constant volumes, and the energy–volume data were fitted to the Murnaghan EOS^30^. First-principles calculations based on DFT were conducted with the PBEsol exchange–correlation functional implemented in Vienna ab-initio Simulation Package^46,47^ to evaluate the phase stabilities of six possible structural types with increasing pressure. The plane wave cutoff energy is set as 520 eV and the integration in the Brillouin zone was performed with 4×4×4 *Γ*-centered *k*-point mesh. On-site Coulomb repulsion *U* of *d* shell of Co ion was set using the Dudarev implementation^3^. The simplest FM order was applied to focus on the pressure effect in all calculations by ignoring the energy shift that complex magnetic orders may lead to. The energy–volume relationship was fitted to the Murnaghan EOS to compute the relative enthalpy and pressure (Δ*H*–*P*) diagram^30^.

### Synthesis

AP-CTO was synthesized via solid-state reaction using stoichiometric mixture of Co_3_O_4_ (99.99%, Aladdin) and TeO_2_ (99.99%, Alfa Aesar)^48^. The starting materials were pelletized and heated in air at 1023 K for 12 h, in which both the heating and cooling rate was set as 5 K/min. Then the asmade AP-CTO precursor was placed in a Pt capsule liner to an Al_2_O_3_ crucible, heated to 1123 K at 5 GPa for 30 min in a Walker-type multi-anvil press before quenched to room temperature. The pressure was then slowly decompressed to ambient. Phase purity was examined by powder XRD measurements in a RIGAKU D-MAX 2200 VPC diffractometer (*λ* = 1.5418 Å). Temperature-dependent NPD measurements were performed at the beamline of a general-purpose powder diffractometer at the China Spallation Neutron Source. SPXD data were collected on beamline BL14B (*λ* = 0.6893 Å) at the Shanghai Synchrotron Radiation Facility. Refinement of the SPXD and NPD data were carried out using programs of Topas-Academic *V*6 and Jana2006, respectively.

### XANES

Both the transmission and fluorescence modes were applied for Co-K XANES data collection with simultaneous standards. The spectra were fitted to linear pre- and post-edge backgrounds and normalized to unity absorption edge stepping across the edge^49–51^. The title compound XANES measurements were conducted at the QAS, 7BM Beamline at Brookhaven National Synchrotron Light Source II (NSLS-II), using a Si(111) channel-cut monochromator in the “qick,” continuous scanning mode. Partial standard spectra were previously collected on beamline X-19A at NSLS-I with a Si-111 double crystal monochromator.

### Magnetic and magnetoelectric measurements

The magnetic properties were measured on a physical property measurement system (PPMS; Quantum Design EverCool-II). PFM observation was carried out by a scanning probe microscopy (MFP-3D, Asylum Research). To improve the PFM sensitivity, a dual frequency resonant-tracking technique (DART, Asylum Research Company) was adopted. The dielectric constant (*ε*) and pyroelectric current (*J*) measurements were performed on HP-CTO, which was mounted on a home-built sample holder, and put into PPMS to control the measuring temperature and magnetic field, using the Alpha-A Analyzer (Novocontrol) and a high-resistance Keithley Model 6517B electrometer, respectively. The pyroelectric current with respect to time was intergrated to achieve the electric polarization (*P*). The permittivity measurements in Fig. 5b were performed on cooling from >100 K with an electric ac field amplitude of 1.5 V/mm. Due to the pyroelectric nature of HP-CTO, shown in Fig. 5c and discussed in the main text, an electric poling field was not required during the initial cooling or the measurements. For the measurements of the polarization in Fig. 5d, the sample was cooled down once to 2 K in zero magnetic field and the magnetic field-dependent measurements were performed by measuring *I* (*H*) without applied electric field for increasing temperatures. The results in Fig. 5e were obtained with the same protocol but in separate cooling cycles.

## DATA AVAILABILITY

All relevant data supporting the findings of this study are available from the corresponding authors upon request. The supporting crystallographic information file may also be obtained from FIZ Karlsruhe, 76344 Eggenstein-Leopoldshafen, Germany (e-mail: crysdata@fiz-karlsruhe.de), on quoting the deposition number CSD-1991205.

## Supplementary Material

Supplementary Material 1

Crystallographic information file

CheckCIF report
