# Supplementary material for: Data-driven computational prediction and experimental realization of exotic perovskite-related polar magnets
Source: npj Quantum Inf. Author manuscript; Available in PMC 2024 Jun 12. (PMC11167729; doi:10.1038/s41535-020-00294-2)
Supplement: Supplementary Material 1 [file NIHMS1918234-supplement-Supplementary_Material_1.docx]

**SUPPLEMENTARY MATERIALS**

Data-driven computational prediction and experimental realization of exotic perovskite-related polar magnets

*Yifeng Han^1,#^ Meixia Wu^1,#^ Churen Gui^2^, Chuanhui Zhu^1^, Zhongxiong Sun^1^, Meihuan Zhao^1^, Aleksandra A. Savina^3^, Artem M. Abakumov^3^, Biao Wang^4^, Feng Huang^4^, LunHua He^5,6,7^, Jie Chen^7,8^, Qingzhen Huang^9^, Mark Croft^10^, Steven Ehrlich^11^, Syed Khalid^11^, Zheng Deng^12^, Changqing Jin^12^, Christoph P. Grams^13^, Joachim Hemberger^13^, Xueyun Wang^14^, Jiawang Hong^14^, Umut Adem^15^, Meng Ye^16^, Shuai Dong^2^, Man-Rong Li^1,*^*

^1^Key Laboratory of Bioinorganic and Synthetic Chemistry of Ministry of Education, School of Chemistry, Sun Yat-Sen University, Guangzhou 510275, China.

^2^School of Physics, Southeast University, Nanjing 211189, China.

^3^Skolkovo Institute of Science and Technology, Bolshoy Boulevard 30, bld. 1, Moscow 121205, Russia.

^4^State Key Laboratory of Optoelectronic Materials and Technologies, School of Materials, Sun Yat-Sen University, Guangzhou 510275, China..

^5^Beijing National Laboratory for Condensed Matter Physics, Institute of Physics, Chinese Academy of Sciences, Beijing 100190, China.

^6^Songshan Lake Materials Laboratory, Dongguan, Guangdong 523808, China.

^7^Institute of High Energy Physics, Chinese Academy of Sciences, Beijing 100049, China.

^8^Spallation Neutron Source Science Center, Dongguan 523803, China.

^9^NIST Center for Neutron Research, National Institute of Standards and Technology, Gaithersburg MD, 20899-6102, USA

^10^Department of Physics, Rutgers, The State University of New Jersey, Piscataway, NJ 08854, USA.

^11^NSLS-II, Brookhaven National Laboratory, Upton, NY, USA.

^12^Institute of Physics, School of Physics, University of Chinese Academy of Sciences, Chinese Academy of Sciences, P. O. Box 603, Beijing 100190, China.

^13^ II Physikalisches Institut, Universität zu Köln, D 50937 Köln, Germany.

^14^School of Aerospace Engineering, Beijing Institute of Technology, Beijing 100081, China.

^15^Department of Materials Science and Engineering, İzmir Institute of Technology, Urla 35430, İzmir, Turkey.

^16^State Key Laboratory of Low Dimensional Quantum Physics and Department of Physics, Tsinghua University, Beijing, 100084, China.

#These authors equally contributed to this work.

Email: [limanrong@mail.sysu.edu.cn](mailto:limanrong@mail.sysu.edu.cn) (M.-R. Li)

**Supplementary Note 1: Data-mining: polymorphs of *A*_2_*BB*’O_6_ with small *A*-site cations**

Exotic double perovskite related *A*_2_*BB*’O_6_ materials with small *A*-site cation, where the ionic radius of *A* is no larger than that of the high spin (HS) Mn^2+^, is an emerging research frontier due to the promising magnetoelectric and polar-structure related properties originated from the magnetic-ion enriched lattices and large structural distortion,^1, 2, 3, 4, 5^ such as the giant magnetoresistance up to +220% in the ferrimagnetic (magnetic Curie temperature *T*_C_ ~ 520 K) half-metal Mn_2_FeReO_6_, ^6, 7^ and above-room-temperature multiferroics in double-LiNbO_3_ structural polar magnet Mn_2_FeMoO_6_ (*T*_C_ ~ 340 K). ^8^ Theoretically, the composition flexibility of exotic *A*_2_*BB*’O_6_ predicts more than 13,000 compounds under charge balance over the periodic table of elements (Note that *A* and *B* can be the same element). However, data-mining of the Inorganic Crystal Structure Database (ICSD) and literature screening reveals only 68 compounds, in which 65 have been experimentally prepared and 3 theoretically predicted (**Table S1**). Therefore, there is still a huge space to explore new materials. Structurally, the 68 compounds can be categorized into six groups: (I) 9 cubic bixbyite-derivatives, (II) 25 rhombohedral corundum-derivatives, (III) 8 Mg_3_TeO_6_-type, (IV) 3 LiSbO_3_-derivatives, (V) 2 *β*-Li_3_VF_6_-type, and (VI) 11 GdFeO_3_-type distorted perovskites.

The crystal structure of cubic bixbyite (*Ia*-3, **Fig. S1a**) is comprised of octahedrally coordinated cations, where *A* and *B* are disordered in (*A*_2/3_*B*_1/3_)O_6_ octahedra to form edge- and corner-sharing framework, in which *B*’O_6_ octahedron is accommodated by sharing all of the 12 edges with the adjacent (*A*_2/3_*B*_1/3_)O_6_ octahedra to form a rather crowded arrangement. The bixbyite-derived Cu_3_WO_6_ (*Pa*-3) comprises the coexistence of CuO_4_, CuO_5_, and CuO_6_ interconnected with WO_6_, and can be regarded as the transitional structure between bixbyite and wolframite.^23, 24, 25^ The rhombohedral conundrum derivative is the most extensively studied family crystallized in centrosymmetric *R*-3*c*, or polar *R*3*c* or *R*3 depending on the cationic ordering degree.^89^ The crystal structure is well exemplified by Ni_3_TeO_6_-type *R*3, showing face-sharing octahedral pairs along *c*-axis and edge-sharing honeycomb-layers in *ab*-plane. (**Fig. S1b**). Mg_3_TeO_6_-type structure (*R*-3) has similar (*A*,*B*)O_6_ octahedral connection with the bixbyite polymorph, while the *B*’O_6_ octahedron is incorporated via sharing half of its edges (6 edges of the opposite oxygen triangle faces) with the neighbor (*A*,*B*)O_6_ (**Fig. S1c**), making the structure less crowded than bixbyite. The LiSbO_3_ derivatives (*Pnn*2, **Fig. S1d**) possess edge-sharing octahedral chains of alternatively ordered *B*O_6_ and *B*’O_6_ with two distinct 6-folded Li atoms zigzagged down the channels. The monoclinic *β*-Li_3_VF_6_ structure (*C*2/*c*, **Fig. S1e**) is less common and only discovered in *A*_3_TeO_6_ (*A* = Zn, ^78^ Co), the rich coordination environment of *A*O_4_, *A*O_5_, *A*O_6_ makes it difficult to sort out the *A* and *B* sites of *A*_2_*BB*’O_6_ form besides the unambiguous *B*’O_6_ motif. The GdFeO_3_-type distorted perovskite structure (*P*2_1_/*n*, **Fig. S1f** has been extensively studied with rock-sort ordering *B*O_6_ and *B*’O_6_ and eight-folded *A*-site coordination.

**Supplementary Note 2: Theoretical calculation**

The energy-volume (*E-V*) curve obtained from Density functional theory (DFT) calculation was fitted to Murnaghan equation of state (EOS) of state:

$$E\left( V \right)=B_{0}V_{0}\left[ \frac{1}{B'(B^{'}-1)}\left( \frac{V_{0}}{V} \right)^{B^{'}-1}+ \frac{V}{B^{'}V_{0}}- \frac{1}{B^{'}-1} \right]+ E_{0}$$

and thus four parameters, *B*_0_ (the bulk modulus), *B*′ (the first derivative of *B*_0_), *V*_0_ (equilibrium volume) and *E*_0_ (equilibrium total energy) at equilibrium state were obtained.

The pressure *P* is derived from the combination of the thermodynamic equation

$$dE=TdS-PdV$$

where *T* is temperature, and Murnaghan EOS, can be described as

$$P= -\left( \frac{\partial E}{\partial V} \right)_{S}= \frac{B_{0}}{B^{'}}[\left( \frac{V_{0}}{V} \right)^{B^{'}}- 1]$$

As well known, the original definition of enthalpy is

$$H=E+PV$$

So the enthalpy-pressure (*H-P­*) relation can be express as

$$H=E_{0}+ \frac{V_{0}\left( P+B_{0} \right)}{B^{'}-1}{(\frac{B_{0}}{B^{'}P+B_{0}})}^{\frac{1}{B'}}- \frac{B_{0}V_{0}}{B^{'}-1}+PV_{0}{(\frac{B_{0}}{B^{'}P+B_{0}})}^{\frac{1}{B'}}$$

**Supplementary Note 3: Crystal structure and phase stability of HP-CTO**

X-ray absorption near edge spectroscopy (XANES) measurements of the K-edges of 3d row transition metals in compounds has proven to be a useful probe of the transition metal valence/configuration. ^90, 91, 92, 93, 94, 95^ These edges are dominated by peak-like 1s to 4p transitions and typically exhibit a chemical shift to higher energies with increasing transition metal valence. The Co-K edge chemical shift to higher energy with increasing valence, between Co^2+^, Co^3+^ and Co^4+^ standard compounds, is illustrated in **Fig. S4a**. The onset of the Co_3_TeO_6_ spectrum in **Fig. S4b** is consistent with a Co^2+^ state in this compound.

The pre-edge portion of the Co-K spectra from **Fig. S4a** are shown in **Fig. S4b**. Such pre-edge spectral features involve quadrupole, and d/p-hybridization induced dipole, transitions into final d-states. Again, an increasing-valence/chemical-shift-to-higher-energy is typically seen in the pre-edge features, along with changes in the spectral distribution. Comparison of the Co_3_TeO_6_ pre-edge feature to those of the standard compounds further supports the Co^2+^ assignment for this compound. Thus, both the main-edge and pre-edge Co-K results support a Co^2+^ state in Co_3_TeO_6_.

**Supplementary Note 4: Magnetic properties and spin structure determination of HP-CTO**

The magnetic structure analysis was carried out with the Jana2006 program. The magnetic structure solution was performed using a formalism of magnetic superspace symmetry. There are three one-dimensional irreducible representations (irreps) for the propagation vector ***k*** = [0, 0, *γ*] in the space group *R*3; the corresponding magnetic superspace groups and their symmetry operators are given in **Table S4**. The magnetization of the atom in the unit cell is therefore defined by the contribution of its magnetic moment in the basic structure ***M***_0_ and the contribution given by a modulation function ***M***(*x*_4_), *x*_4_ = ***k***(***T*** + ***r***), where *x*_4_ is the so-called internal coordinate in a (3+1)-dimensional space, ***T*** is the lattice translation of the nuclear structure, and ***r*** is the position of the atom in the unit cell of the nuclear structure. Thus, the magnetic moment of the atom is expressed as:

***M*** = ***M***_0_ + ***M****_s_*sin(*2*π*x*_4_) + ***M****_c_*cos(2π*x*_4_)

where ***M****_s_* and ***M****_c_* denote the sine and cosine Fourier first-order coefficients. No higher-order harmonic terms are required as only the first-order magnetic satellites are observed in the NPD patterns at both temperatures.

All three magnetic superspace groups were tested in the refinement. It was not possible to properly index the magnetic satellites in the sp. gr. *R*3.1’(00*γ*)0*s*, thus it was ruled out. In the *R*3.1’(00*γ*)*ts* (or its enantiomorphic twin *R*3.1’(00*γ*)-*ts*) model, there are three magnetic symmetrically inequivalent atoms Co1, Co2, Co3, all on the 3*a* positions of the *R*3 space group. The magnetic superspace group *R*3.1’(00*γ*)*ts* implies the following restriction on the Fourier coefficients of Co^2+^ magnetic moments located at the 3*a* position:

These restrictions imply that the ***M****_s_*[Co] and ***M****_c_*[Co] vectors are perpendicular to each other and they have the same length so they describe a screw-type circular ordering. The solution was found with the following restrictions on the coefficients of the magnetic moment modulation functions (not imposed by symmetry):

*M*_s_*_x_*[Co1]= *M*_s_*_x_*[Co2]; *M*_s_*_x_*[Co3] = -*M*_s_*_x_*[Co2]; *M*_s_*_y_*[Co1] = *M*_s_*_y_*[Co2] = *M*_s_*_y_*[Co3] = 0

Thus, in the final model, only one magnetic amplitude *M*_s_*_x_*[Co1] was refined. The magnetic moment parameters were calculated and the obtained results are summarized in **Table S5**. The resulting fit provides good agreement with the experimental data from both high-Q and medium-Q databanks, as shown in **Fig. 4a**. The details of the refined parameters and reliability factors are given in **Tables S6** and **S8**. Co_3_TeO_6_ adopts a constant moment spiral magnetic structure consisting of antiferromagnetic helixes propagating along the *с* axis. The helixes are associated with the Co1, Co2 and Co3 atomic chains (**Fig. 4b**). The magnetic moment of the Co atoms is constant being equal to 2.97(1) *μ_B_* at *T* = 5 K and 2.25(1) *μ_B_* at *T* = 45 K. At *T* = 5 K the magnetic moments of Co1 chain is rotated about ≈ 53.0° with respect to that of Co2, and about ≈ 85.6° with respect to Co3 (**Fig. 5a**). The phase shift *φ* between the Co1 and Co2 helixes is 0, while between the Co1 and Co3 helixes is *π*.

**Supplementary Note 5: Magnetoelectric properties of HP-CTO**

The temperature dependence of d*M*/d*T* (the thermal derivative of the magnetization) and *ε*′ (the dielectric constant) curves are compared over the same temperature range for values of the applied field *H*: between of 0.0 and 0.5 T (**Fig. S10a**); and for *H* between 1.5 and 7 T (**Fig. S10b**). These plots emphasize the close magnetoelectric coupling of the polarization and magnetization order parameters in HP-CTO. In **Fig. S10a** similar critical signature (a positive peak) in both the d*M*/d*T* and *ε*′ curves yield values for the low-field transition temperature into the AFM-LF-1 (antiferromagnetic-low-field phase 1) just below *T* = 58 K. Moreover, they are similarly in quantitative agreement about the decrease in this ordering temperature in the *H* = 0 to 0.5 T range. The field dependent transition temperatures in this low-field range are plotted in the magnetoelectric phase diagram in **Fig. 5g** in the main-body of the text. It should also be noted in the 17-24 K temperature range: that more subtle structures are also visible in the d*M*/d*T* and *ε*′ curves; and that moreover the thermal magnetization curves for *H* = 0.1 and 0.5 T (see **Fig. 3b**) exhibit coupled peak-type/nonlinear downturn structures in the same temperature range. This would appear to be quite consistent with the neutron scattering measurements determination of a transition into a lower temperature AFM-LF-2 phase (antiferromagnetic-low-field phase 2) in this temperature range. The onset of this AFM-LF-2 phase is indicated by a dashed box in the figure above and in the magnetoelectric phase diagram in **Fig. 5g**. **Fig. S10b** manifests similar critical signatures (positive peaks) in both the d*M*/d*T* and *ε*′ curves yield for fields between 1.5 and 5 T indicate a transition into a higher magnetization antiferromagnetic high field (AFM-HF) state for which the transition temperature is systematically shifted downward with increasing field. (It should be noted the 5 T, *ε*′ data terminates before the transition temperature that is visible in the d*M*/d*T* curve.) The locus of the *ε*′ and d*M*/d*T* determined transition temperatures into the AFM-HF state are shown in **Fig. 5g** again underscore the close agreement between the magnetic/dielectric transition temperatures and critical signatures should be noted. The *H* = 7 T d*M*/d*T* curve gives no clear signature of a phase transition and the extrapolation of the transition temperatures for the AFM-HF state in the phase diagram indicate the suppression just above *H* =5 T.

all of the magnetization curves and is indicated in the **Fig. 5g** phase diagram by gray bars. The increasing length of the gray bars indicates the broadening of this magnetization increase. In **Fig. S10b** the same effect is replicated in the d*M*/d*T* curve and the trend is indicated by a gray arrow. Interestingly there is a similar systematic shift in the dielectric constant- curves in the same temperature/field range which is also highlighted in the figure with a gray arrow. The signature the canted-correlations in the *ε*′ curves is not precisely the same as in the d*M*/d*T* curves but the trend is similar thereby supporting a continued magnetoelectric coupling. The change in the detailed magnetic/dielectric response is not unexpected since correlations in this higher temperature/field range are canted moment like whereas in the lower temperature transitions they were AF-like.

**Supplementary Note 6: Magnetic field variation measurements.**

Inset of **Fig. 3c** in the main text showing the detailed cross over of the field induced transition from 1^st^ order-hysteretic at low *T*, to continuous at higher temperatures. Note: the *M*(*H*) curve at *T* = 65 K is above the transition; the *T* = 58 K curve is barely above the transition; and that both are nonlinear but do not exhibit a field induced transition. **Fig. S11a** shows the comparison of the field dependence of the magnetization *M* (*H*) and the polarization *P* (*H*) loops at *T* = 2 K for fields between 0 and 14 T. The low-field hysteresis at the first order AFM-LF2/AFM-HF transition is clear in both the *M*(*H*) and *P*(*H*) measurements. Moreover, there is a nonlinear crossover in the field dependence of both in the vicinity. It should be recalled that *H* = 5 T was the high field stability limit of the AFM-HF phase indicated by the thermal measurements in **Fig. S10b**. **Fig. S11b** shows the magnetization loop at *T* = 2 K from: *H* = 0 to 14 T (*M*_u_), black dashed curve; *H* = 14 to 0 T (*M*_d_), solid red curve. The hysteretic low field first-order transition between the AFM-LF and AFM-HF phases is clear. The magnitude of the numerical derivative d*M*_d_/d*H* (right scale), dotted red curve, is also shown in the figure and exhibits a dramatic peak illustrating the decreasing field AFM-HF to AFM-LF instability. The high data density, with appropriate spline smoothing, allows the identification of more subtle features in the d*M*_d_/d*H* curve. Specifically, a weak peak near *H* = 5 T a subtle peak is visible. In the Inset of the figure the d*M*_d_/d*H* curve in the vicinity of 5 T is expanded and further smoothed (heavy red line). In the smoothed curve in the inset clearly supports the presence critical (albeit subtle) transition peak in d*M*_d_/d*H* near *H* =5 T. This peak is highly consistent with the high-field and stability limit of the AFM-HF phase.

Supplementary Figures


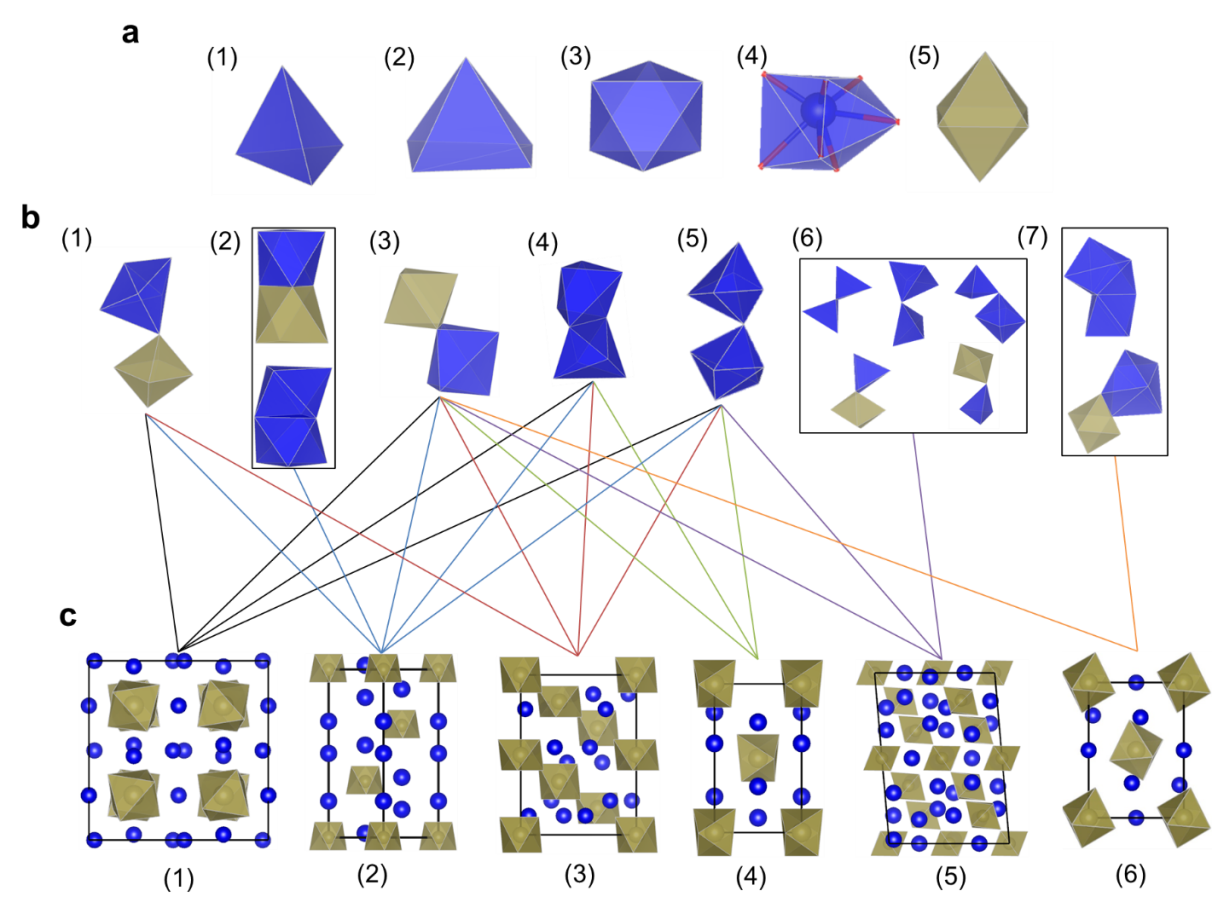


**Fig. S1** **Crystal structures of the six possible polymorphs of exotic *A*_2_*BB*’O_6_ from ICSD and literature screening.** (**a**) Basic coordination units of cationic sites: (1) tetrahedron, (2) distorted rectangular pyramid, (3) octahedron, (4) 8-folded, and (5) octahedron, where deep blue and clay polyhedra are for *A*/*B* and *B*’ polyhedra, respectively. (**b**) Structure motifs: (1) corner-sharing tetrahedron and octahedron, (2) face- and edge-sharing octahedral pairs, (3) corner-sharing octahedra, (4) edge-sharing tetrahedron and octahedron, (5) corner-sharing distorted rectangular pyramid and octahedron, (6) corner-sharing tetrahedron-tetrahedron, tetrahedron-distorted rectangular pyramid, tetrahedron-octahedron pairs, and (7) edge-sharing distorted rectangular pyramid and octahedron. (**c**) Crystal structures and corresponding structural motifs: (1) cubic bixbyite-derivatives (*Ia*-3), (2) rhombohedral corundum-derivatives (*R*3, *R*-3, or *R*3*c*), (3) Mg_3_TeO_6_-type (*R*-3), (4) LiSbO_3_-derivatives (*Pnn*2), (5) *β*-Li_3_VF_6_-type (*C*2/*c*), and (6) GdFeO_3_-type distorted perovskites (*P*2_1_/*n*). Only the *B*’O_6_ are shown as polyhedron for clarity.

**Fig. S2 Comparison of the XRD patterns of CTO** **synthesized at ambient pressure and 5 GPa.** (**a**) AP (*C*2/*c*) and (**b**) 5 GPa (*R*3).


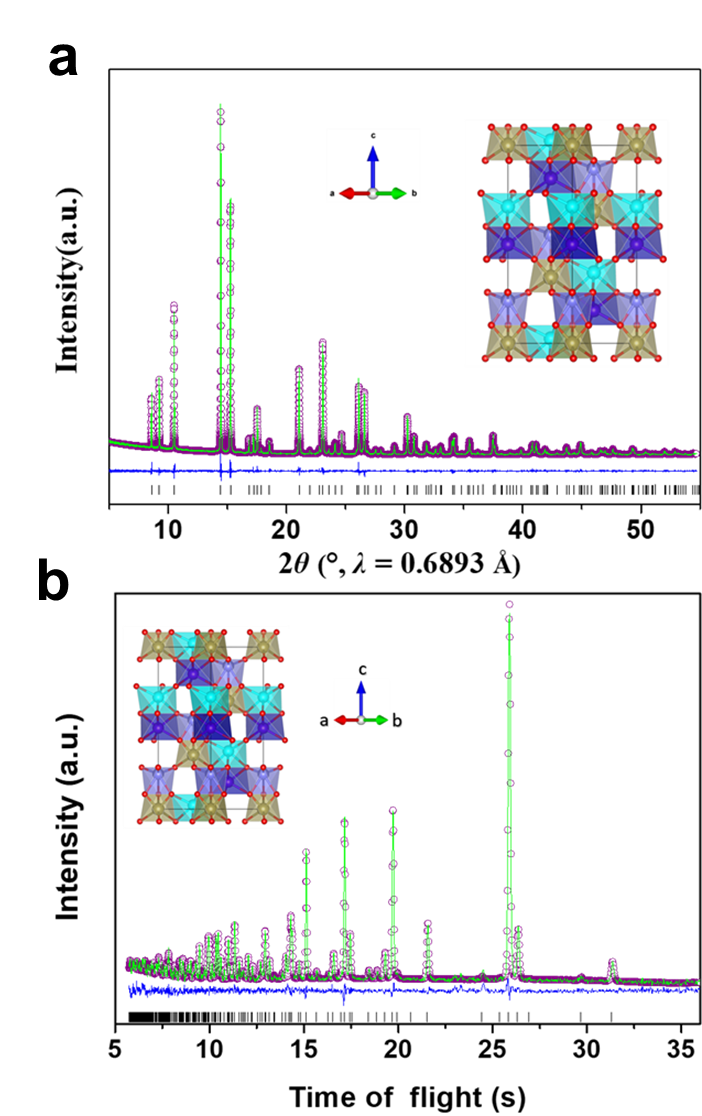


**Fig. S3 Rietveld refinement of the (a) SPXD and (b) NPD data for HP-CTO.** The purple circle represents the calculated fit, the green line the observed data, the deep blue line the difference, black tick marks the peak positions of HP-CTO. Inset shows the crystal structure viewed along [110] direction. Co1/Co2/Co3/Te, purple, cyan, and brown spheres; CoO_6_ octahedra, purple, cyan and lilac; TeO_6_ octahedra, brown; O, red spheres.


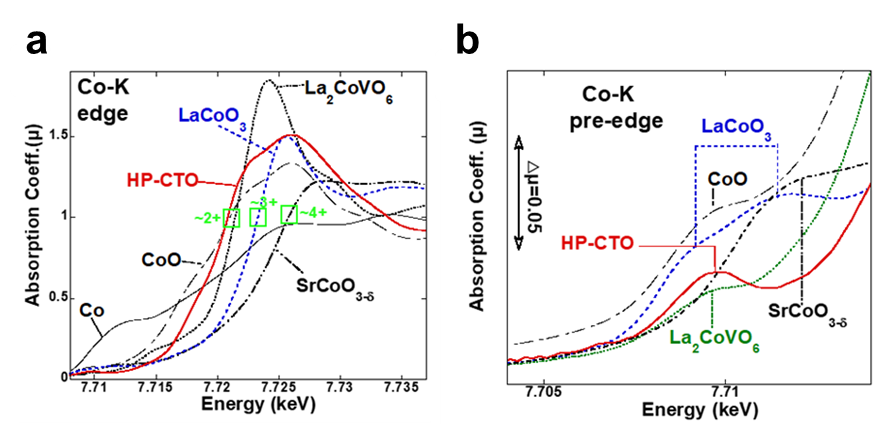


**Fig. S4** **XANES analysis of HP-CTO.** (**a**) The Co-K main-edge is compared to the edges of a series of octahedrally coordinated standard compounds with differing formal valence states: Co^2+^, CoO (with edge sharing) La_2_CoVO_6_ (a corner sharing double-perovskite); Co^3+^, LaCoO_3_ (a corner sharing perovskite); and Co~^4+^, SrCoO_3-_*_δ_* (a corner sharing perovskite). (**b**) The Co-K pre-edge compared to the same series of standard compounds as in the previous figure.


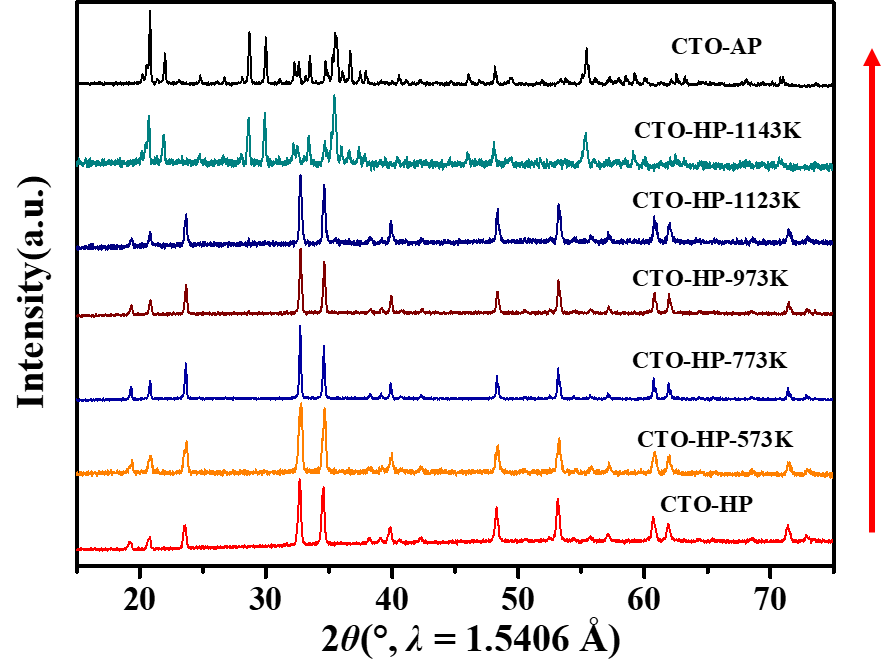


**Fig. S5** **Phase stability of HP-CTO examined by XRD after annealing between 300 and 1143 K for 30 min in Ar atmosphere at each temperature point.** The XRD pattern of the AP-CTO is added on the top for comparison. The relative intensity variation of the peak after annealing between 300 and 1128 K is caused by preferred orientation.


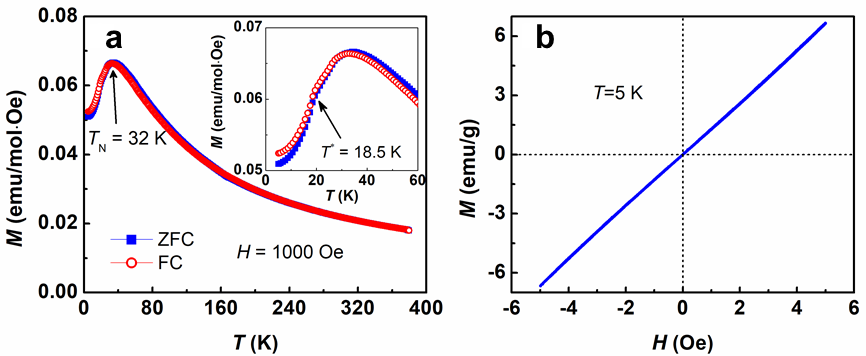


**Fig. S6** **Magnetic properties of the AP-CTO.** (a) ZFC-FC curves of AP-CTO, (b) *M*-*H* curve of AP-CTO at 5 K.


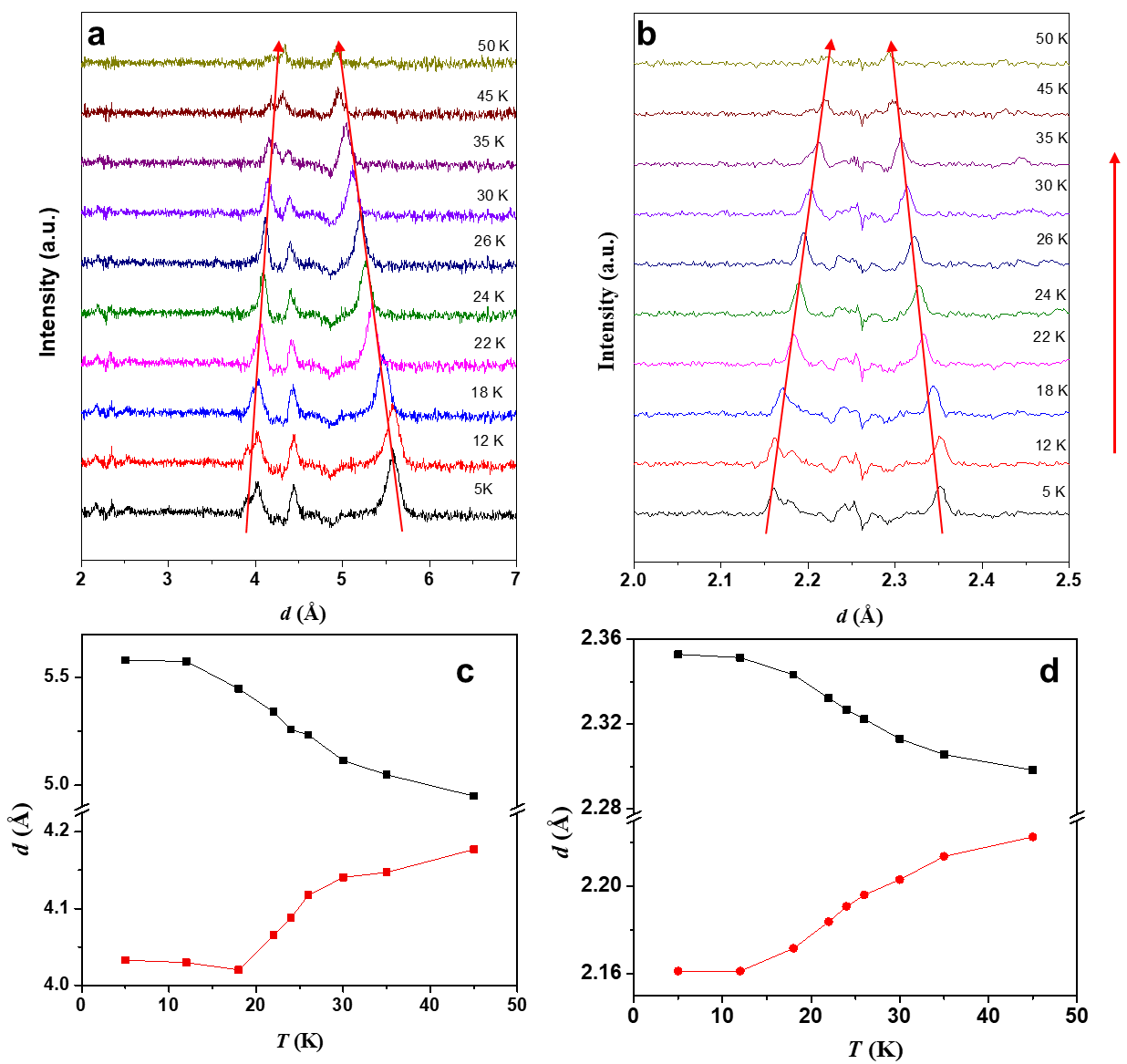


**Fig. S7** **The difference magnetic satellite curves between low temperature (below 55 K) and 55 K.** (**a**) bank 3; (**b**) bank 2; (**c**) and (**d**) illustrate the peak shift with temperature for bank 3 and bank 2, respectively. Magnetic peak data were obtained by subtracting the signals of nuclear structure. Red arrows show the temperature increasing trend.


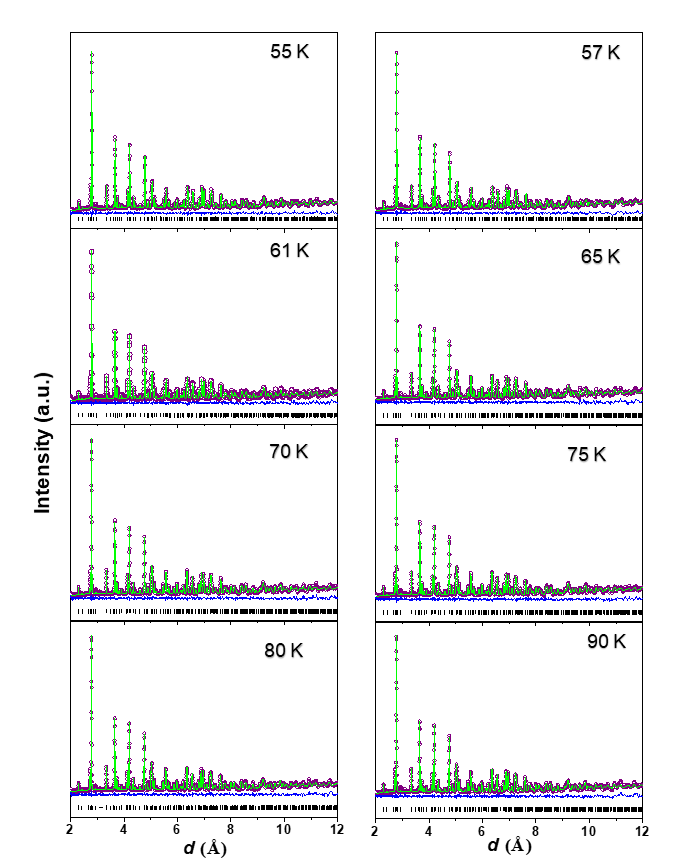


**Fig. S8** **Refinements of the NPD data of HP-CTO between 55 and 90 K** **using the high-resolution bank 2.** Experimental, calculated, and difference curves are shown as purple, green, and blue, respectively. The black bars mark the reflection positions for the nuclear reflections.


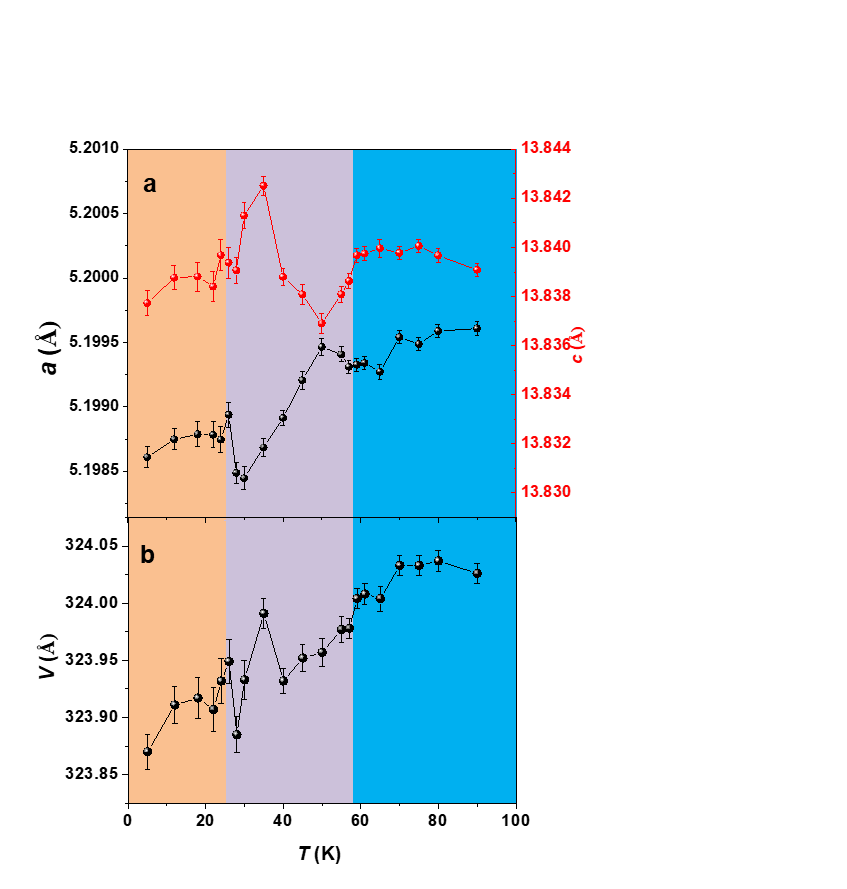


**Fig. S9** **Lattice parameter evolution of HP-CTO between 5 and 90 K extracted from the *in situ* variable temperature NPD data.** (**a**) *a* and *c* *vs* *T* plots; (**b**) *V* *vs* *T* plot. The orange, light purple, and blue background are corresponding to the temperature regions below *T*_1_ (24 K), between *T*_1_ and *T*_2_ (58 K), and above *T*_2_, respectively.


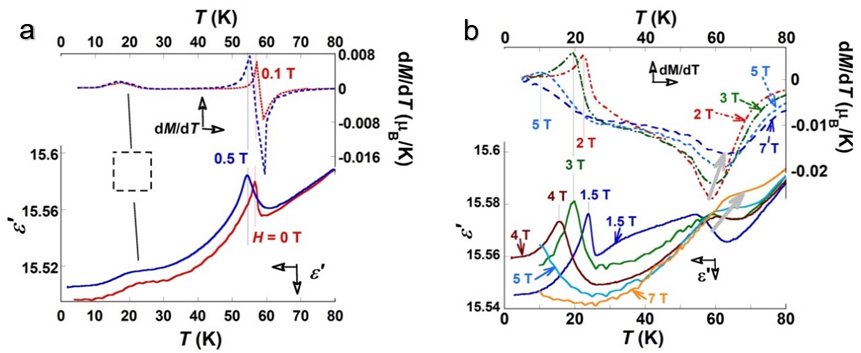


**Fig. S10 Comparison of the temperature dependence of d*M*/d*T* and *ε*′ curves of HP-CTO**. **(a)** at low magnetic fields**; (b)** at high magnetic fields.

The d*M*/d*T* curves shown in the inset of d*M*/d*T* curve **Fig. 3b** all indicate a negative peak structure which broadens and moves to higher temperature with increasing *H*. While not a phase transition of this d*M*/d*T* peak reflects the broad increase of canted AF-correlations which carry a net moment in the 60-65 K range in


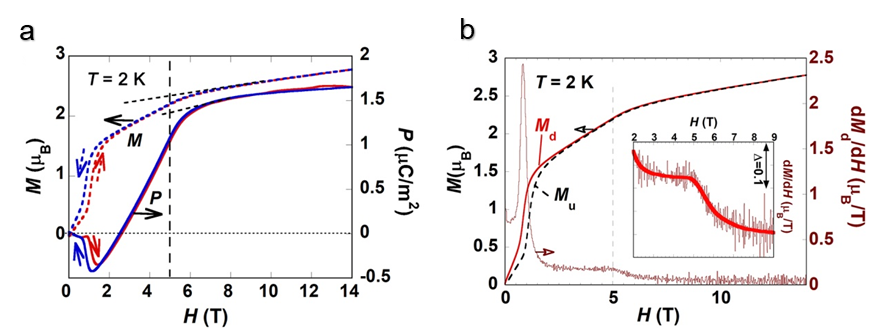


**Fig. S11 Magnetoelctric coupling analysis of HP-CTO at 2 K**. **(a)** Comparison of *M*-*H* and *P*-*H* at *T* = 2 K; **(b)** *M*-*H* curve and its d*M*/d*H* at 2 K.

**Supplementary Tables**

**Table S1** Structural polymorphs, synthesis conditions, and reported physical properties of the known *A*_2_*BB*’O_6_ compounds with small *A*-site cations from literature screening and data-mining of the Inorganic Crystal Structure Database (ICSD).

| **Compound** | **Synthesis Conditions** | **Reported Physical Properties** | **Ref.** |
| --- | --- | --- | --- |
| **I. Bixbyite-derivatives, cubic** | | | |
| ***Ia*-3** | | | |
| Cu_2_CoTeO_6_ | 850 ℃, AP*^a^* | - | ^9, 10^ |
| Cu_2_NiTeO_6_ | 800 ℃, AP | - | ^9^ |
| Cu_2_FeSbO_6_ | 950 ℃, AP | AFM*^b^*, *T*_N_*^c^* = 15.6 K | ^11^ |
| Cu_2_GaSbO_6_ | 950 ℃, AP | - | ^11^ |
| Cu_2_MnSbO_6_ | 950 ℃, AP | AFM, *T*_N_ = 5.9 K | ^11^ |
| Cu_3_TeO_6_ | 500~600 ℃, AP | AFM, *T*_N_ = 60 K | ^12, 13, 14, 15, 16, 17, 18, 19, 20, 21^ |
| In_2_RuFeO_6_ | 1300 ℃, AP | AFM, *T*_N_ = 90 K | ^22^ |
| In_2_RuMnO_6_ | 1400 ℃, AP | AFM, *T*_N_ < 41 K | ^22^ |
| ***Pa*-3** | | | |
| Cu_3_WO_6_ | 800 ℃, AP | Quasistatic spin freezing at 7.0 K | ^23, 24, 25^ |
| **II. Corundum-derivatives, rhombohedral** | | | |
| **LiNbO_3_-type, *R*3*c*** | | | |
| Mn_2_FeNbO_6_ | 1300 ℃, 7 GPa | Multiferroic, *T*_N_ = 90 K, *P*_S_*^d^* = 32 μC/cm^2^ | ^26^ |
| Mn_2_FeTaO_6_ | 1300 ℃, 7 GPa | Multiferroic, *T*_N_ = 80 K, *P*_S_ = 23 μC/cm^2^ | ^26^ |
| Zn_2_FeTaO_6_ | 1350 ℃, 9 GPa | Multiferroic, *T*_N_ = 22 K, *P*_S_ = 50 μC/cm^2^ | ^27^ |
| **Ilmenite, *R*-3** | | | |
| Mn_2_CrSbO_6_ | 600 ℃, AP | AFM, *T*_N_ = 60 K | ^28, 29^ |
| Mn_2_FeSbO_6_ | 900 ℃, 3 GPa | FiM,*^e^* *T*_C_*^f^* = 270 K | ^28, 30, 31, 32, 33^ |
| Mn_2_AlSbO_6_ | 900 ℃, 6 GPa | - | ^28, 29^ |
| Mn_2_GaSbO_6_ | 900 ℃, 6 GPa | AFM, *T*_N_ = 50 K | ^28, 29^ |
| **Ordered ilmenite, *R*3** | | | |
| Li_2_GeTeO_6_ | 750 ℃, AP | - | ^34^ |
| Mn_2_FeMoO_6_ | Annealed at AP | Multiferroic, *T*_C_ = 229 K, *P*_S_ = 55 μC/cm^2^ | ^35^ |
| Mn_2_InSbO_6_ | 1100 ℃, 5 GPa | AFM, *T*_N_ = 38 K | ^36^ |
| **Ni_3_TeO_6_-type, *R*3** | | | |
| Li_2_ZrTeO_6_ | 700 ℃, AP | SHG*^g^* with high laser-damage threshold | ^37, 38^ |
| Li_2_HfTeO_6_ | 700 ℃, AP | - | ^37^ |
| Mn_2_ScSbO_6_ | 1250 ℃, 5.5 GPa | FM, *T*_C_ = 42 K | ^39, 40^ |
| Mn_2_FeMoO_6_ | 1350 ℃, 8 GPa | Multiferroic, *T*_C_ = 340 K, *P*_S_ = 68 μC/cm^2^ | ^8^ |
| Mn_2_FeWO_6_ | 1400 ℃, 8 GPa | Multiferroic, *T*_N_ = 70 K, *P*_S_ = 59.5 μC/cm^2^ | ^41^ |
| Mn_2_MnWO_6_ | 1400 ℃, 8 GPa | Magnetoelectric, *T*_N_ = 58 K, *P*_S_ = 63 μC/cm^2^ | ^42^ |
| Mn_2_ScNbO_6_ | 1200 ℃, 6 GPa | SHG, FiM, *T*_C_ = 53 K | ^43^ |
| Mn_2_ScTaO_6_ | 1200 ℃, 6 GPa | SHG, FiM, *T*_C_ = 50 K | ^25^ |
| Ni_3_TeO_6_ | 700 ℃, AP | Magnetoelectric, *T*_N_ = 52 K | ^16, 17, 44, 45, 46, 47, 48, 49, 50, 51, 52^ |
| Ni_2_ScSbO_6_ | 1300 ℃, AP | - | ^39^ |
| Ni_2_InSbO_6_ | 1300 ℃, AP | FM, *T*_N_ = 74 K | ^39^ |
| Co_3_TeO_6_ | 850 ℃, 5 GPa | Multiferroic, *T*_N_ = 58 K, *P*_S_ = 57 μC/cm^2^ | This work |
| Zn_2_FeOsO_6_ | Predicted | Magnetoelectric, *T*_C_ = 394 K | ^53^ |
| Sc_2_FeMoO_6_ | Predicted | Room-temepratrue FiM, *P*_S_ = 7.1 μC/cm^2^ | ^54^ |
| Sc_2_FeMoO_6_ | Predicted | Room-temepratrue FiM, *P*_S_ = 8.7 μC/cm^2^ | ^54^ |
| **III. Mg_3_TeO_6_-type, rhombohedral, *R*-3** | | | |
| Mg_3_TeO_6_ | 800 ℃, AP | - | ^48, 55, 56, 57, 58^ |
| Mn_3_TeO_6_ | 830 ℃, AP | Multiferroic,*T*_N_ = 23 K | ^17, 59, 60, 61, 62^ |
| Mn_3_WO_6_ | AP, Laser technique | - | ^63^ |
| Mn_2_CdTeO_6_ | 900 ℃, 2.67 MPa | - | ^60^ |
| Mn_2_InSbO_6_ | 1300 ℃, AP | Short-range magnetic order | ^64^ |
| Mn_2_ScSbO_6_ | 1300 ℃, AP | Short-range magnetic order | ^64^ |
| Sc_3_CrO_6_ | 1100 ℃, AP | - | ^65^ |
| Sc_3.31_Al_0.69_O_6_ | 1500 ℃, AP | - | ^66^ |
| **IV. LiSbO_3_-derivatives, orthorhombic, *Pnn*2** | | | |
| Li_2_TiTeO_6_ | 700 ℃, AP | - | ^67^ |
| Li_2_SnTeO_6_ | 700 ℃, AP | - | ^67^ |
| Li_2_GeTeO_6_ | 800 ℃, 5 GPa | SHG | ^68^ |
| **V. *β*-Li_3_VF_6_-type, monoclinic, *C*2/*c*** | | | |
| Co_3_TeO_6_ | 700 ℃, AP | Multiferroic, *T*_N_ = 26 K | ^17, 59, 60, 69, 70, 71, 72, 73, 74, 75, 76, 77^ |
| Zn_3_TeO_6_ | 750 ℃, AP | - | ^78^ |
| **VI. GdFeO_3_-type distorted perovskites, *P*2_1_/*n 11*** | | | |
| In_2_NiMnO_6_ | 1327 ℃, 6 GPa | AFM, *T*_N_ = 26 K | ^79^ |
| Sc_2_NiMnO_6_ | 1500 ℃, 6 GPa | Magnetodielectric, AFM, *T*_N_ = 35 K | ^80^ |
| Mn_3_TeO_6_ | 800 ℃, 5 GPa | Magnetodielectric, AFM, *T*_N_ = 37 K | ^81^ |
| Mn_2_CrSbO_6_ | 900 ℃, 6 GPa | AFM, *T*_N_ = 55 K | ^29^ |
| Mn_2_FeSbO_6_ | 1000 ℃, 6 GPa | AFM, *T*_N_ = 19.5 K | ^3, 5, 28, 31, 32, 82, 83^ |
| Mn_2_CoReO_6_ | 1300 ℃, 8 GPa | AFM, *T*_N_ = 94 K | ^84^ |
| Mn_2_FeReO_6_ | 1350 ℃, 5-11 GPa | FiM half-metal, *T*_C_ = 520 K | ^6, 7^ |
| Mn_2_MnReO_6_ | 1400 ℃, 5-8 GPa | AFM semiconductor, *T* = 110 K | ^85, 86^ |
| Mn_2_ScSbO_6_ | 1200 ℃, 12 GPa | AFM, *T*_N_ = 22.3 K | ^40, 64^ |
| Mn_2_VSbO_6_ | 1200 ℃, 6 GPa | Spin-glass transition at 9.5 K | ^87^ |
| Mn_2_Fe_0.8_Mo_1.2_O_6_ | 1350 ℃, 8 GPa | FiM semiconductor, *T*_C_ = 194 K | ^88^ |

*^a^*AP = ambient pressure; *^b^*AFM = antiferromagnetic; *^c^T*_N_ = Néel temperature; *^d^P*_S_ = spontaneous polarization, *^e^*FiM = ferrimagnetic, *^f^T*_C_ = magnetic Curie temperature, *^g^*SHG = second harmonic generation.

| **Table S2a.** Refined structural parameters in HP-CTO from SPXD data collected at room temperature. | | | | | |
| --- | --- | --- | --- | --- | --- |
| **atom** | site | *x* | *y* | *z* | *B* (Å^2^) |
| **Co1** | 3*a* | 0 | 0 | 0.3688(1) | 0.93(3) |
| **Co2** | 3*a* | 0 | 0 | 0.6490(1) | 0.73(3) |
| **Co3** | 3*a* | 0 | 0 | 0.8609(1) | 0.81(3) |
| **Te** | 3*a* | 0 | 0 | 0.16064(7) | 0.60(1) |
| **O1** | 9*b* | 0.287(2) | -0.005(2) | 0.2474(6) | 1.19(5) |
| **O2** | 9*b* | 0.669(1) | -0.022(2) | 0.7566(6) | 1.19(5) |
| Rhombohedral, space group *R*3 (no. 146), *a* = 5.19148(6) Å, *c* = 13.8216(1) Å, *V* = 322.606(9) Å^3^, *Z* = 3, *R*_wp_ = 7.90%, *R*_p_ = 5.87%. | | | | | |

| **Table S2b** Refined structural parameters in HP-CTO from NPD data collected at room temperature. | | | | | |
| --- | --- | --- | --- | --- | --- |
| **atom** | site | *x* | *y* | *z* | *B* (Å^2^) |
| **Co1** | 3*a* | 0 | 0 | 0.3652(9) | 0.71(7) |
| **Co2** | 3*a* | 0 | 0 | 0.6470(9) | 0.71(7) |
| **Co3** | 3*a* | 0 | 0 | 0.859 (1) | 0.71(7) |
| **Te** | 3*a* | 0 | 0 | 0.1589(7) | 1.43(9) |
| **O1** | 9*b* | 0.2870(8) | -0.005(1) | 0.2461(2) | 1.04(4) |
| **O2** | 9*b* | 0.6657(8) | -0.0333(7) | 0.7533(2) | 1.06(5) |
| Rhombohedral, space group *R*3 (no. 146), *a* = 5.189(3) Å, *c* = 13.811(7) Å, *V* = 322.1(4) Å^3^, *Z* = 3, *R*_wp_ = 6.80%, *R*_p_ = 7.42%. | | | | | |

**Table S3a** Selected interatomic distances (Å), bond angles (º), bond valence sums (BVS) calculations, and octahedral distortion parameters (*∆*) in the crystal structure HP-CTO from SPXD at room temperature.

| Co1O_6_ |  | Co2O_6_ |  |
| --- | --- | --- | --- |
| Co1-O | 1.999(12) ×3 | Co2-O | 2.063(12) ×3 |
|  | 2.255(10) ×3 |  | 2.230(8) ×3 |
| <Co1-O> | 2.12(1) | <Co2-O> | 2.14(1) |
| BVS | 1.96 | BVS | 1.80 |
| Δ_Co_(×10^-4^) | 40.5 | Δ_Co_(×10^-4^) | 17.6 |
| O-Co1-O | 70.7(4) | O-Co2-O | 80.4(3) |
|  | 87.7(4) |  | 86.8(4) |
|  | 89.0(3) |  | 90.2(3) |
|  | 106.7(3) |  | 100.7(3) |
|  | 154.0(4) |  | 165.2(3) |
| Co3O_6_ |  | TeO_6_ |  |
| Co3-O | 2.010(7) ×3 | Te-O | 1.925(10) ×3 |
|  | 2.200(8) ×3 |  | 1.947(7) ×3 |
| <Co3-O> | 2.11(1) | <Te-O> | 1.93(1) |
| BVS | 2.03 | BVS | 5.70 |
| Δ_Co_(×10^-4^) | 18.2 | Δ_Te_(×10^-4^) | 0.8 |
| O-Co3-O | 78.3(3) | O-Te-O | 85.3(4) |
|  | 81.7(3) |  | 86.8(3) |
|  | 89.3(4) |  | 90.3(5) |
|  | 107.4(3) |  | 97.0(3) |
|  | 159.1(4) |  | 171.3(4) |

**Table S3b** Selected interatomic distances (Å), bond angles (º), bond valence sums (BVS) calculations, and octahedral distortion parameters (*∆*) in the crystal structure HP-CTO from NPD at room temperature.

| Co1O_6_ |  | Co2O_6_ |  |
| --- | --- | --- | --- |
| Co1-O | 2.045(6) ×3 | Co2-O | 2.058(8) ×3 |
|  | 2.22(1) ×3 |  | 2.212(9) ×3 |
| <Co1-O> | 2.13(1) | <Co2-O> | 2.13(1) |
| BVS | 1.87 | BVS | 1.85 |
| Δ_Co_(×10^-4^) | 17.8 | Δ_Co_(×10^-4^) | 13.0 |
| O-Co1-O | 71.5 (4) | O-Co2-O | 80.7(4) |
|  | 87.3(2) |  | 87.3(1) |
|  | 88.1(2) |  | 88.9(1) |
|  | 107.2(4) |  | 101.1(4) |
|  | 154.1(6) |  | 165.3(6) |
| Co3O_6_ |  | TeO_6_ |  |
| Co3-O | 2.013(6) ×3 | Te-O | 1.926(6) ×3 |
|  | 2.21(1) ×3 |  | 1.926(8) ×3 |
| <Co3-O> | 2.11(1) | <Te-O> | 1.926(7) |
| BVS | 2.00 | BVS | 5.85 |
| Δ_Co_(×10^-4^) | 21.7 | Δ_Te_(×10^-4^) | - |
| O-Co3-O | 77.7(2) | O-Te-O | 85.1(4) |
|  | 80.9(4) |  | 87.1(1) |
|  | 90.8(2) |  | 91.9(2) |
|  | 107.3(4) |  | 95.6(4) |
|  | 158.1(6) |  | 171.8(4) |
|  | | | |

**Table S4** Magnetic irreducible representations of the parent space group *R* 3 for the incommensurate propagation vector ***k*** = [0, 0, *γ*] and symmetry operators (−*m* means a ‘time inversion’ operation, while *m* is an operation without time inversion).

| **Irrep** | \| **Shubnikov superspace group** \| \| --- \| | **Symmetry operators** | | | | | |
| --- | --- | --- | --- | --- | --- | --- | --- | --- |
| \| *mΛ*_1_*Λ*_1_ \| \| --- \| | \| *R*3.1’(00*γ*)0*s* \| \| --- \| | *E* | *x*_1_ | *x*_2_ | *x*_3_ | *x*_4_ | *m* |
|  |  | 3 | *-x*_2_ | *x*_1_*-x*_2_ | *x*_3_ | *x*_4_ | *m* |
|  |  | 3^2^ | *-x*_1_ *+ x*_2_ | *-x*_1_ | *x*_3_ | *x*_4_ | *m* |
|  |  | *E(1*’*\|*00½*)* | *x*_1_ | *x*_2_ | *x*_3_ | *x*_4_*+*½ | *-m* |
|  |  | 3(1’\|00½) | *-x*_2_ | *x*_1_*-x*_2_ | *x*_3_ | *x*_4_*+*½ | *-m* |
|  |  | 3^2^(1’\|00½) | *-x*_1_ *+ x*_2_ | *-x*_1_ | *x*_3_ | *x*_4_*+*½ | *-m* |
| *mΛ*_3_*Λ*_3_ | *R*3.1*’(00γ)ts* | *E* | *x*_1_ | *x*_2_ | *x*_3_ | *x*_4_ | *m* |
|  |  | 3 | *-x*_2_ | *x*_1_*-x*_2_ | *x*_3_ | *x*_4_*+*⅓ | *m* |
|  |  | 3^2^ | *-x*_1_ *+ x*_2_ | *-x*_1_ | *x*_3_ | *x*_4_*+*⅔ | *m* |
|  |  | *E(*1’*\|*00½*)* | *x*_1_ | *x*_2_ | *x*_3_ | *x*_4_*+*½ | *-m* |
|  |  | 3(1’\|00½) | *-x*_2_ | *x*_1_*-x*_2_ | *x*_3_ | *x*_4_*+*⅚ | *-m* |
|  |  | 3^2^(1’\|00½) | *-x*_1_ *+ x*_2_ | *-x*_1_ | *x*_3_ | *x*_4_*+*⅟_6_ | *-m* |
| *mΛ*_2_*Λ*_2_ | *R*3.1*’(00γ)-ts* | *E* | *x*_1_ | *x*_2_ | *x*_3_ | *x*_4_ | *m* |
|  |  | 3 | *-x*_2_ | *x*_1_*-x*_2_ | *x*_3_ | *x*_4_*+*⅔ | *m* |
|  |  | 3^2^ | *-x*_1_ *+ x*_2_ | *-x*_1_ | *x*_3_ | *x*_4_*+*⅓ | *m* |
|  |  | *E(1*’*\|*00½*)* | *x*_1_ | *x*_2_ | *x*_3_ | *x*_4_*+*½ | *-m* |
|  |  | 3(1’\|00½) | *-x*_2_ | *x*_1_*-x*_2_ | *x*_3_ | *x*_4_*+*⅟_6_ | *-m* |
|  |  | 3^2^(1’\|00½) | *-x*_1_ *+ x*_2_ | *-x*_1_ | *x*_3_ | *x*_4_*+*⅚ | *-m* |

**Table S5** Magnetic ordering parameters of HP-CTO at different temperatures with the superspace group *R*3.1’(00*γ*)*ts* with the magnetic modulation vector **k** = [0, 0, *γ*].

| **Atom** | ***M***_1_*_sx_* | ***M***_1_*_sy_* | ***M***_1_*_cx_* | ***M***_1_*_cy_* | **\|M\|** (*μ_B_*) |
| --- | --- | --- | --- | --- | --- |
| 5 K | | | | | |
| Co1 | 2.97(2) | 0 | 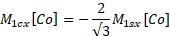 | 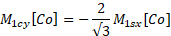 | 2.97(1) |
| Co2 | ***M***_1_*_sx_*[*Co*1] | 0 |  |  |  |
| Co3 | ***-M***_1_*_sx_*[*Co*1] | 0 |  |  |  |
| 45 K | | | | | |
| Co1 | 2.25(3) | 0 | 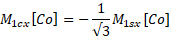 | 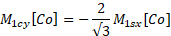 | 2.25(1) |
| Co2 | ***M***_1_*_sx_*[*Co*1] | 0 |  |  |  |
| Co3 | -***M***_1_*_sx_*[*Co*1] | 0 |  |  |  |

| **Table S6** Refined structural parameters in CTO-HP from NPD collected at 5 K. | | | | |
| --- | --- | --- | --- | --- |
| **atom** | site | *x* | *y* | *z* |
| **Co1** | 3*a* | 0 | 0 | 0.3653(10) |
| **Co2** | 3*a* | 0 | 0 | 0.6453(9) |
| **Co3** | 3*a* | 0 | 0 | 0.8636(10) |
| **Te** | 3*a* | 0 | 0 | 0.1643(6) |
| **O1** | 9*b* | 0.2890(13) | -0.0070(16) | 0.2527(8) |
| **O2** | 9*b* | 0.6667(11) | -0.0354(11) | 0.7595(8) |
| Rhombohedral, space group *R* 3 (no. 146), *a* = 5.19859(9) Å, *c* = 13.8380(4) Å, *V* = 323.87(1) Å^3^, *Z* = 3, *R*_wp_ = 7.43%, *R*_p_ = 7.25%. | | | | |

**Table S7** Selected interatomic distances (Å), bond angles (º), bond valence sums (BVS) calculations, and octahedral distortion parameters (*∆*) in the crystal structure HP-CTO from NPD at 5 K.

| Co1O_6_ |  | Co2O_6_ |  |
| --- | --- | --- | --- |
| Co1-O | 2.092(9) ×3 | Co2-O | 1.998(8) ×3 |
|  | 2.17(1) ×3 |  | 2.28(1) ×3 |
| <Co1-O> | 2.13(1) | <Co2-O> | 2.14(1) |
| BVS | 1.84 | BVS | 1.92 |
| Δ_Co_(×10^-4^) | 3.52 | Δ_Co_(×10^-4^) | 42.7 |
| O-Co1-O | 74.3(3) | O-Co2-O | 78.3(2) |
|  | 87.5(2) |  | 86.9(2) |
|  | 88.8(2) |  | 87.8(2) |
|  | 104.9(2) |  | 104.3(3) |
|  | 157.8(2) |  | 160.3(2) |
| Co3O_6_ |  | TeO_6_ |  |
| Co3-O | 2.02(1) ×3 | Te-O | 1.922(8) ×3 |
|  | 2.18(1) ×3 |  | 1.95(1) ×3 |
| <Co3-O> | 2.10(1) | <Te-O> | 1.93(1) |
| BVS | 2.03 | BVS | 5.70 |
| Δ_Co_(×10^-4^) | 14.5 | Δ_Te_(×10^-4^) | 1.07 |
| O-Co3-O | 78.2(2) | O-Te-O | 84.9(3) |
|  | 81.4(2) |  | 86.8(3) |
|  | 91.1(2) |  | 91.9(3) |
|  | 106.3(3) |  | 95.8(2) |
|  | 159.2(2) |  | 171.4(3) |
|  | | | |

| **Table S8** Structural parameters of HP-CTO refined from NPD data collected at 45 K. | | | | |
| --- | --- | --- | --- | --- |
| **atom** | site | *x* | *y* | *z* |
| **Co1** | 3*a* | 0 | 0 | 0.3685(9) |
| **Co2** | 3*a* | 0 | 0 | 0.6492(9) |
| **Co3** | 3*a* | 0 | 0 | 0.8626(10) |
| **Te** | 3*a* | 0 | 0 | 0.1626(6) |
| **O1** | 9*b* | 0.2907(10) | -0.0056(12) | 0.2501(5) |
| **O2** | 9*b* | 0.6677(9) | -0.0352(9) | 0.7577(5) |
| Rhombohedral, space group *R*3 (no. 146), *a* = 5.19905(16) Å, *c* = 13.8397(6) Å, *V* = 323.97(2) Å^3^, *Z* = 3, *R*_wp_ = 6.74%, *R*_p_ = 6.60%. | | | | |

**Table S9** Selected interatomic distances (Å), bond angles (º), bond valence sums (BVS) calculations, and octahedral distortion parameters (∆) in the crystal structure of HP-CTO refined from NPD collected at 45 K.

| Co1O_6_ |  | Co2O_6_ |  |
| --- | --- | --- | --- |
| Co1-O | 2.068(8) ×3 | Co2-O | 2.039(9) ×3 |
|  | 2.24(1) ×3 |  | 2.22 (1) ×3 |
| <Co1-O> | 2.15(1) | <Co2-O> | 2.13(1) |
| BVS | 1.76 | BVS | 1.89 |
| Δ_Co_(×10^-4^) | 14.5 | Δ_Co_(×10^-4^) | 18.0 |
| O-Co1-O | 72.3(5) | O-Co2-O | 79.5(5) |
|  | 87.0(3) |  | 88.0(3) |
|  | 88.2(3) |  | 88.6(3) |
|  | 106.9(5) |  | 101.6(5) |
|  | 154.9(6) |  | 164.1(7) |
| Co3O_6_ |  | TeO_6_ |  |
| Co3-O | 2.011(7) ×3 | Te-O | 1.948(9) ×3 |
|  | 2.19(1) ×3 |  | 1.926(7) ×3 |
| <Co3-O> | 2.10(1) | <Te-O> | 1.937(9) |
| BVS | 2.04 | BVS | 5.68 |
| Δ_Co_(×10^-4^) | 18.3 | Δ_Te_(×10^-4^) | 0.03 |
| O-Co3-O | 78.1(3) | O-Te-O | 86.4(3) |
|  | 80.9(5) |  | 85.4(4) |
|  | 90.9(3) |  | 91.9(3) |
|  | 106.9(5) |  | 95.9(4) |
|  | 158.5(7) |  | 171.6(5) |
|  | | | |

|  | **55 K** | **57 K** | **59 K** | **61 K** | **65 K** | **70 K** | **75 K** | **80 K** | **90 K** |
| --- | --- | --- | --- | --- | --- | --- | --- | --- | --- |
| ***a* (Å)** | 5.19940(5) | 5.19931(5) | 5.19932(5) | 5.19934(5) | 5.19923(5) | 5.19954(5) | 5.19948(5) | 5.19958(5) | 5.19961(5) |
| ***c* (Å)** | 13.8381(3) | 13.8386(3) | 13.8396(2) | 13.8397(2) | 13.8400(2) | 13.8397(2) | 13.8400(2) | 13.8396(2) | 13.8390(2) |
| **Co1-*z*** | 0.374(1) | 0.375(1) | 0.3746(9) | 0.375(1) | 0.3769(9) | 0.376(1) | 0.376(1) | 0.3777(9) | 0.377(1) |
| **Co1-*Biso* (Å^2^)** | 0.9(2) | 0.9(2) | 0.5(1) | 0.7(1) | 0.4(1) | 0.8(1) | 1.3(2) | 0.6(1) | 0.9(2) |
| **Co2-*z*** | 0.663(3) | 0.661(2) | 0.657(1) | 0.661(2) | 0.660(1) | 0.658(1) | 0.658(1) | 0.662(1) | 0.659(1) |
| **Co2-*Biso* (Å^2^)** | 2.5(3) | 2.4(3) | 1.1(2) | 2.1(3) | 1.0(2) | 1.0(2) | 0.7(2) | 1.6(1) | 1.0(2) |
| **Co3-*z*** | 0.872(1) | 0.868(1) | 0.870(1) | 0.871(1) | 0.870(1) | 0.868(1) | 0.868(1) | 0.867(1) | 0.866(1) |
| **Co3-*Biso* (Å^2^)** | 0.7(2) | 1.1(2) | 2.2(3) | 1.5(3) | 1.2(2) | 1.6(3) | 1.2(2) | 1.0(2) | 1.5(3) |
| **Te-*z*** | 0.1679(9) | 0.1666(6) | 0.1694(7) | 0.1678(3) | 0.1685(7) | 0.1691(6) | 0.1682(6) | 0.1686(7) | 0.1678(6) |
| **Te-*Biso* (Å^2^)** | 1.2(1) | 0.93(9) | 1.2(1) | 0.82(9) | 1.3(1) | 1.18(9) | 1.1(1) | 1.13(9) | 1.1(1) |
| **O1-x** | 0.2854(9) | 0.2854(8) | 0.2876(8) | 0.2883(8) | 0.2848(8) | 0.2868(8) | 0.2837(8) | 0.2853(8) | 0.2870(1) |
| **O1-y** | -0.005(1) | -0.004(1) | -0.0033(9) | -0.0026(9) | -0.006(1) | -0.0041(9) | -0.008(1) | -0.007(1) | -0.0046(9) |
| **O1-z** | 0.2574(3) | 0.2586(3) | 0.2583(3) | 0.2586(3) | 0.2581(3) | 0.2581(3) | 0.2583(3) | 0.2580(3) | 0.2586(3) |
| **O1-*Biso* (Å^2^)** | 1.23(6) | 1.39(5) | 1.43(6) | 1.21(5) | 1.10(5) | 1.26(5) | 1.18(5) | 1.18(5) | 1.19(5) |
| **O2-*x*** | 0.6679(8) | 0.6677(7) | 0.6680(8) | 0.6677(8) | 0.6676(8) | 0.6672(8) | 0.6656(8) | 0.6676(8) | 0.6675(8) |
| **O2-*y*** | -0.0317(7) | -0.0321(6) | -0.0334(6) | -0.0331(6) | -0.0310(7) | -0.0330(6) | -0.0321(7) | -0.0322(7) | -0.0329(7) |
| **O2-*z*** | 0.7648(3) | 0.7654(3) | 0.7651(3) | 0.7651(3) | 0.7651(3) | 0.7655(2) | 0.7661(3) | 0.7654(2) | 0.7655(3) |
| **O2-*Biso* (Å^2^)** | 0.88(4) | 0.83(4) | 0.82(4) | 0.86(4) | 0.98 (5) | 0.84(4) | 0.92(4) | 0.95(5) | 0.95(5) |
| ***R*_wp_** | 5.78 | 5.44 | 6.37 | 6.42 | 6.51 | 6.17 | 6.13 | 6.13 | 6.15 |
| ***R*_p_** | 6.35 | 6.00 | 7.24 | 7.32 | 7.18 | 6.89 | 6.77 | 6.97 | 6.90 |

**Table S10** Crystal **s**tructure parameters in HP-CTO refined from NPD data collected at 55 - 90 K.

# Supplementary References

1. Giaquinta DM, zur Loye H-C. Structural predictions in the *AB*O_3_ phase diagram. *Chem. Mater.* **6**, 365-372 (1994).

2. Navrotsky A. Energetics and crystal chemical systematics among ilmenite, lithium niobate, and perovskite structures. *Chem. Mater.* **10**, 2787-2793 (1998).

3. Vasala S, Karppinen M. *A*_2_*B*'*B*''O_6_ perovskites: A review. *Prog. Solid. State. Chem.* **43**, 1-36 (2015).

4. Hossain A, Bandyopadhyay P, Roy S. An overview of double perovskites *A*_2_*B*'*B*''O_6_ with small ions at *A* site: Synthesis, structure and magnetic properties. *J. Alloys. Compd.* **740**, 414-427 (2018).

5. Alexei AB, Wei Y. High-pressure synthesis, crystal chemistry and physics of perovskites with small cations at the A site. *J. Phys. Condens. Matter.* **26**, 163201 (2014).

6. Arévalo-López AM, McNally GM, Attfield JP. Large magnetization and frustration switching of magnetoresistance in the double-perovskite ferrimagnet Mn_2_FeReO_6_. *Angew. Chem. Int. Ed.* **54**, 12074-12077 (2015).

7. Li M-R*, et al.* Giant magnetoresistance in the half-metallic double-perovskite ferrimagnet Mn_2_FeReO_6_. *Angew. Chem. Int. Ed.* **54**, 12069-12073 (2015).

8. Li M-R*, et al.* Magnetic-structure-stabilized polarization in an above-room-temperature ferrimagnet. *Angew. Chem. Int. Ed.* **53**, 10774-10778 (2014).

9. Wedel B, Kimio I, Sugiyama K. Crystal structure of dicopper nickel hexaoxotellurate, Cu_2_NiTeO_6_. *Z. Kristallogr. NCS.* **216**, 345-346 (2001).

10. Becker R, Berger H. Cu_2_CoTeO_6_. *Acta Crystallogr. E* **62**, i261-i262 (2006).

11. Bazuev GV, Golovkin BG, Zubkov VG, Tyutyunnik AS. Synthesis, crystal structure, and magnetic properties of complex oxides Cu_2_BSbO_6_ (B = Mn, Fe, Ga) with a bixbyite structure. *J. Solid. State. Chem.* **113**, 132-137 (1994).

12. Falck L, Lindqvist O, Moret J. Tricopper(II) tellurate(VI). *Acta Crystallogr. B* **34**, 896-897 (1978).

13. Herak M*, et al.* Novel spin lattice in Cu_3_TeO_6_ : an antiferromagnetic order and domain dynamics. *J. Phys. Condens. Matter.* **17**, 7667 (2005).

14. Choi KY, Lemmens P, Choi ES, Berger H. Lattice anomalies and magnetic excitations of the spin web compound Cu_3_TeO_6_. *J. Phys. Condens. Matter.* **20**, 505214 (2008).

15. M H. Cubic magnetic anisotropy of the antiferromagnetically ordered Cu_3_TeO_6_. *Solid. State. Commun.* **151**, 1588-1592 (2011).

16. Kaleva G*, et al.* Phase transitions of (Cu,Ni)_3_TeO_6_ solid solutions. *Inorg. Mater.* **47**, 1132-1140 (2011).

17. Mathieu R, Ivanov SA, Nordblad P, Weil M. Enhancement of antiferromagnetic interaction and transition temperature in *M*_3_TeO_6_ systems (*M* = Mn, Co, Ni, Cu). *Eur. Phys. J. B.* **86**, 1-4 (2013).

18. Månsson M, *et al*. Magnetic order and transitions in the spin-web compound Cu_3_TeO_6_. *Phys. Procedia.* **30**, 142-145 (2012).

19. He Z, Itoh M. Magnetic behaviors of Cu_3_TeO_6_ with multiple spin lattices. *J. Magn. Magn. Mater.* **354**, 146-150 (2014).

20. Zhu X, Wang Z, Su X, Vilarinho PM. New Cu_3_TeO_6_ ceramics: phase formation and dielectric properties. *ACS Appl. Mater. Interfaces.* **6**, 11326-11332 (2014).

21. Caimi G, Degiorgi L, Berger H, Forró L. Optical evidence for a magnetically driven structural transition in the spin web Cu_3_TeO_6_. *EPL (Europhysics Letters)* **75**, 496 (2006).

22. de la Calle C, *et al*. Structure and magnetic properties of In_2_RuMnO_6_ and In_2_RuFeO_6_: Heavily transition-metal doped In_2_O_3_ -type bixbyites. *Solid. State. Commun.* **152**, 95-99 (2012).

23. Gebert E, Kihlborg L. The crystal structure of a new copper wolfram oxide, Cu_3_WO_6_. *Acta Chem. Scand.* **23**, 221-231 (1969).

24. Hase M, Uchinokura K. Spin-singlet ground state with energy gap in Cu_3_WO_6_: A new kind of an RVB state? *Physica. B* **215**, 325-328 (1995).

25. Fudamoto Y*, et al.* Muon spin relaxation in the spin-ring system Cu_3_WO_6_: Quasistatic spin freezing at 7.0 K. *Phys. Rev. B* **65**, 174428 (2002).

26. Li M-R*, et al.* Polar and magnetic Mn_2_Fe*M*O_6_ (*M*=Nb, Ta) with LiNbO_3_-type structure: high-pressure synthesis. *Angew. Chem. Int. Ed.* **52**, 8406-8410 (2013).

27. Li M-R*, et al.* Designing polar and magnetic oxides: Zn_2_FeTaO_6_ - in search of multiferroics. *J. Am. Chem. Soc.* **136**, 8508-8511 (2014).

28. Bazuev GV, *et al*. High pressure synthesis and polymorphism of complex oxides Mn_2_*B*SbO_6_ (*B*= Fe, V, Cr, Ga, Al). *J. Solid. State. Chem.* **124**, 333-337 (1996).

29. Dos santos-Garcia AJ, *et al*. Synthesis, structures and magnetic properties of the dimorphic Mn_2_CrSbO_6_ oxide. *Dalton. Trans.* **44**, 10665-10672 (2015).

30. Mathieu R*, et al.* Magnetic order near 270 K in mineral and synthetic Mn_2_FeSbO_6_ ilmenite. *Appl. Phys. Lett.* **98**, 202505 (2011).

31. Hudl M, *et al*. Investigation of the magnetic phase transition and magnetocaloric properties of the Mn_2_FeSbO_6_ ilmenite. *J. Magn. Magn. Mater.* **331**, 193-197 (2013).

32. Mathieu R*, et al.* Mn_2_FeSbO_6_: A ferrimagnetic ilmenite and an antiferromagnetic perovskite. *Phys. Rev. B* **87**, 014408 (2013).

33. Dos santos-García AJ*, et al.* Large magnetoelectric coupling near room temperature in synthetic melanostibite Mn_2_FeSbO_6_. *Angew. Chem. Int. Ed.* **56**, 4438-4442 (2017).

34. Woodward PM, Sleight AW, Du L-S, Grey CP. Structural studies and order–disorder phenomenon in a series of new quaternary tellurates of the type *A*^2+^*M*^4+^Te^6+^O_6_ and *A*^1+2^*M*^4+^Te^6+^O_6_. *J. Solid. State. Chem.* **147**, 99-116 (1999).

35. Li M-R*, et al.* Low-temperature cationic rearrangement in a bulk metal oxide. *Angew. Chem. Int. Ed.* **55**, 9862-9867 (2016).

36. Arévalo-López ÁM*, et al.* Evolving spin periodicity and lock-in transition in the frustrated ordered ilmenite-type Mn_2_InSbO_6_. *Phys. Rev. B* **98**, 214403 (2018).

37. Choisnet J, Rulmont A, Tarte P. Les tellurates mixtes Li_2_ZrTeO_6_ et Li_2_HfTeO_6_: un nouveau phénomène d'ordre dans la famille corindon. *J. Solid. State. Chem.* **75**, 124-135 (1988).

38. Lu W*, et al.* Rational design of a LiNbO_3_-like nonlinear optical crystal, Li_2_ZrTeO_6_, with high laser-damage threshold and wide mid-IR transparency window. *J. Am. Chem. Soc.* **140**, 13089-13096 (2018).

39. Ivanov SA*, et al.* Spin and dipole ordering in Ni_2_InSbO_6_ and Ni_2_ScSbO_6_ with corundum-related structure. *Chem. Mater.* **25**, 935-945 (2013).

40. Solana-Madruga E*, et al.* High pressure synthesis of polar and non-polar cation-ordered polymorphs of Mn_2_ScSbO_6_. *Dalton. Trans.* **44**, 20441-20448 (2015).

41. Li M-R*, et al.* Mn_2_FeWO_6_: A new Ni_3_TeO_6_-type polar and magnetic oxide. *Adv. Mater.* **27**, 2177-2181 (2015).

42. Li M-R*, et al.* Magnetostriction-polarization coupling in multiferroic Mn_2_MnWO_6_. *‎Nat. Commun.* **8**, 2037 (2017).

43. Feng HL*, et al.* High-pressure synthesis and ferrimagnetism of Ni_3_TeO_6_-type Mn_2_Sc*M*O_6_ (*M* = Nb, Ta). *Inorg. Chem.* **58**, 15953-15961 (2019).

44. Becker R, Berger H. Reinvestigation of Ni_3_TeO_6_. *Acta Crystallogr. E* **62**, i222-i223 (2006).

45. Wang XY, *et al*. Interlocked chiral/polar domain walls and large optical rotation in Ni_3_TeO_6_. *APL Mater.* **3**, 076105 (2015).

46. Wu F, Kan E, Tian C, Whangbo M-H. Theoretical analysis of the spin exchange and magnetic dipole−dipole interactions leading to the magnetic structure of Ni_3_TeO_6_. *Inorg. Chem.* **49**, 7545-7548 (2010).

47. Živković I, Prša K, Zaharko O, Berger H. Ni_3_TeO_6_ -a collinear antiferromagnet with ferromagnetic honeycomb planes. *J. Phys. Condens. Matter.* **22**, 056002 (2010).

48. Blasse G, Hordijk W. The vibrational spectrum of Ni_3_TeO_6_ and Mg_3_TeO_6_. *J. Solid. State. Chem.* **5**, 395-397 (1972).

49. Newnham RE, Meagher EP. Crystal structure of Ni_3_TeO_6_. *Mater. Res. Bull.* **2**, 549-554 (1967).

50. Zupan J, Kolar D, Urbanc V. Magnetic properties of Ni_3_TeO_6_. *Mater. Res. Bull.* **6**, 1353-1359 (1971).

51. Oh YS*, et al.* Non-hysteretic colossal magnetoelectricity in a collinear antiferromagnet. *‎Nat. Commun.* **5**, 3201 (2014).

52. Kim JW*, et al.* Successive magnetic-field-induced transitions and colossal magnetoelectric effect in Ni_3_TeO_6_. *Phys. Rev. Lett.* **115**, 137201 (2015).

53. Wang PS, Ren W, Bellaiche L, Xiang HJ. Predicting a ferrimagnetic phase of Zn_2_FeOsO_6_ with strong magnetoelectric coupling. *Phys. Rev. Lett.* **114**, 147204 (2015).

54. Song G, Zhang W. Comparative studies on the room-temperature ferrielectric and ferrimagnetic Ni_3_TeO_6_-type *A*_2_FeMoO_6_ compounds (*A* = Sc, Lu). *Sci. Rep.* **6**, 20133 (2016).

55. Schulz H, Bayer G. Structure determination of Mg_3_TeO_6_. *Acta Crystallogr. B* **27**, 815-821 (1971).

56. Newnham RE, Dorrian JF, Meagher EP. Crystal structure of Mg_3_TeO_6_. *Mater. Res. Bull.* **5**, 199-202 (1970).

57. Schulz H, Bayer G. A new structure type Mg_3_TeO_6_. *Naturwissenschaften.* **57**, 393-393 (1970).

58. Kasper H. Spektralphotometrische Ermittlung der Koordinationsverhältnisse in Kristallgittern. III. Über Magnesiumtellurat Mg_3_TeO_6_ und Magnesiumindium-antimonat Mg_2_InSbO_6_. *Z. Anorg. Allg. Chem.* **356**, 329-336 (1968).

59. Golubko NV*, et al.* Synthesizing and investigating the structure and phase transitions in *A*_3_TeO_6_ (*A* - Mn, Co, Ni) oxides. *Bull. Russ. Acad. Sci.: Phys.* **74**, 724-726 (2010).

60. Singh H*, et al.* Structural investigations on Co_3-_*_x_*Mn*_x_*TeO_6_; (0 < *x* ≤ 2); High temperature ferromagnetism and enhanced low temperature anti-ferromagnetism. *J. Appl. Phys* **116**, 074904-074901-074904-074909 (2014).

61. Weil M. Mn_3_TeO_6_. *Acta Crystallogr. E* **62**, i244-i245 (2006).

62. Zhao L*, et al.* Mn_3_TeO_6_ – a new multiferroic material with two magnetic substructures. *Phys. Status Solidi* **9**, 730-734 (2015).

63. Klüver E, Müller-Buschbaum H. Ein neues Mangan(II)-Oxowolframat: Mn_3_WO_6_. *Z. Anorg. Allg. Chem.* **620**, 733-736 (1994).

64. Ivanov S, *et al*. Short-range spin order and frustrated magnetism in Mn_2_InSbO_6_ and Mn_2_ScSbO_6_. *Eur. J. Inorg. Chem.* **2011**, 4691-4699 (2011).

65. Todorov ND, *et al*. Raman spectroscopy and lattice-dynamical calculations of Sc_3_CrO_6_ single crystals. *Phys. Rev. B* **85**, 214301 (2012).

66. Müller D, Assenmacher W, Mader W. Darstellung und Struktur von Sc_3_(Sc_0. 31_Al_0. 69_)O_6_ - einem neuen Abkömmling des Mg_3_TeO_6_-Strukturtyps. *Z. Anorg. Allg. Chem.* **630**, 2483-2489 (2004).

67. Choisnet J, Rulmont A, Tarte P. Ordering phenomena in the LiSbO_3_ type structure: The new mixed tellurates Li_2_TiTeO_6_ and Li_2_SnTeO_6_. *J. Solid. State. Chem.* **82**, 272-278 (1989).

68. Zhao M-H*, et al.* Reversible structural transformation between polar polymorphs of Li_2_GeTeO_6_. *Inorg. Chem.* **58**, 1599-1606 (2019).

69. Becker R, Johnsson M, Berger H. A new synthetic cobalt tellurate: Co_3_TeO_6_. *Acta Crystallogr. C* **62**, i67-i69 (2006).

70. Singh H, *et al*. Structural study in ceramic multiferroic Co_3_TeO_6_ and analysis of possible Co-Co networks. *AIP. Conf. Proc.* **1665**, 060021 (2015).

71. Singh H, *et al*. Short range ferromagnetic, magneto-electric, and magneto-dielectric effect in ceramic Co_3_TeO_6_. *J. Appl. Phys* **119**, 044104 (2016).

72. Singh H, *et al*. Observation of high-spin mixed oxidation state of cobalt in ceramic Co_3_TeO_6_. *J. Appl. Phys* **116**, 214106-214101-214106-214107 (2014).

73. Harris AB. Symmetry analysis of multiferroic Co_3_TeO_6_. *Phys. Rev. B* **85**, 100403 (2012).

74. Her JL*, et al.* Magnetic phase diagram of the antiferromagnetic cobalt tellurate Co_3_TeO_6_. *Phys. Rev. B* **84**, 235123 (2011).

75. Li W-H*, et al.* Interplay between the magnetic and electric degrees of freedom in multiferroic Co_3_TeO_6_. *Phys. Rev. B* **85**, 094431 (2012).

76. Tolédano P*, et al.* First-order multi-k phase transitions and magnetoelectric effects in multiferroic Co_3_TeO_6_. *Phys. Rev. B* **85**, 214439 (2012).

77. Wang C-W*, et al.* Complex magnetic couplings in Co_3_TeO_6_. *Phys. Rev. B* **88**, 184427 (2013).

78. Weil M. Zn_3_TeO_6_. *Acta Crystallogr. E* **62**, i246-i247 (2006).

79. Yi W, *et al*. High-pressure synthesis, crystal structure, and properties of In_2_NiMnO_6_ with antiferromagnetic order and field-induced phase transition. *Inorg. Chem.* **52**, 14108-14115 (2013).

80. Yi W*, et al.* Sc_2_NiMnO_6_: A double-perovskite with a magnetodielectric response driven by multiple magnetic orders. *Inorg. Chem.* **54**, 8012-8021 (2015).

81. Su H-P*, et al.* Predicted polymorph manipulation in an exotic double perovskite oxide. *J. Mater. Chem. C* **7**, 12306-12311 (2019).

82. Santos-García AJD, Ritter C, Solana-Madruga E, Sáez-Puche R. Magnetic and crystal structure determination of Mn_2_FeSbO_6_ double perovskite. *J. Phys. Condens. Matter.* **25**, 206004 (2013).

83. Tyutyunnik AP, Bazuev GV, Kuznetsov MV, Zainulin YG. Crystal structure and magnetic properties of double perovskite Mn_2_FeSbO_6_. *Mater. Res. Bull.* **46**, 1247-1251 (2011).

84. Frank CE*, et al.* Mn_2_CoReO_6_: a robust multisublattice antiferromagnetic perovskite with small *A*-site cations. *Chem. Commun.* **55**, 3331-3334 (2019).

85. Arevalo-Lopez AM, Stegemann F, Attfield JP. Competing antiferromagnetic orders in the double perovskite Mn_2_MnReO_6_ (Mn_3_ReO_6_). *Chem. Commun.* **52**, 5558-5560 (2016).

86. Li M-R*, et al.* Mn_2_MnReO_6_: synthesis and magnetic structure determination of a new transition-metal-only double perovskite canted antiferromagnet. *Chem. Mater.* **28**, 3148-3158 (2016).

87. Bazuev GV, Tyutyunnik AP, Kuznetsov MV, Zainulin YG. Structural, magnetic, and XPS studies of the double-perovskite Mn_2_VSbO_6_. *J. Supercond. Nov. Magn.* **31**, 2907-2914 (2018).

88. Li M-R*, et al.* Mn_2_(Fe_0.8_Mo_0.2_)MoO_6_: A double perovskite with multiple transition metal sublattice magnetic effects. *Chem. Mater.* **30**, 4508-4514 (2018).

89. Cai G-H, Greenblatt M, Li M-R. Polar magnets in double corundum oxides. *Chem. Mater.* **29**, 5447-5457 (2017).

90. Huang Y-H, *et al*. Double-perovskite anode materials Sr_2_*M*MoO_6_ (*M* = Co, Ni) for solid oxide fuel cells. *Chem. Mater.* **21**, 2319-2326 (2009).

91. Kayser P*, et al.* Crystal and magnetic structure of Sr_2_*B*IrO_6_ (*B* = Sc, Ti, Fe, Co, In) in the framework of multivalent iridium double perovskites. *Eur. J. Inorg. Chem.* **2015**, 5027-5038 (2015).

92. Mandal TK, *et al*. La_2_MnVO_6_ double perovskite: a structural, magnetic and X-ray absorption investigation. *J. Mater. Chem.* **19**, 4382-4390 (2009).

93. Poltavets VV, Croft M, Greenblatt M. Charge transfer, hybridization and local inhomogeneity effects in Na*_x_*CoO_2_∙*_y_*H_2_O: An x-ray absorption spectroscopy study. *Phys. Rev. B* **74**, 125103 (2006).

94. Ramanujachary K, *et al*. Substitutional effects of 3*d* transition metals on the magnetic and structural properties of quasi-two-dimensional La_5_Mo_4_O_16_. *J. Solid. State. Chem.* **164**, 60-70 (2002).

95. Sunstrom IV JE, Ramanujachary K, Greenblatt M, Croft M. The synthesis and properties of the chemically oxidized perovskite, La_1–_*_x_*Sr*_x_*CoO_3−_*_δ_* (0.5≤ *x* ≤ 0.9). *J. Solid. State. Chem.* **139**, 388-397 (1998).

# 
