# Supplementary material for: Data-driven computational prediction and experimental realization of exotic perovskite-related polar magnets
Source: npj Quantum Inf. Author manuscript; Available in PMC 2024 Jun 12. (PMC11167729; doi:10.1038/s41535-020-00294-2)
Supplement: CheckCIF report [file NIHMS1918234-supplement-CheckCIF_report.pdf]

checkCIF (basic structural check) running

Checking for embedded fcf data in CIF ...
No extractable fcf data in found in CIF

checkCIF/PLATON (basic structural check)

You have not supplied any structure factors. As a result the full set of tests cannot be run.

THIS REPORT IS FOR GUIDANCE ONLY. IF USED AS PART OF A REVIEW PROCEDURE FOR PUBLICATION, IT SHOULD NOT REPLACE THE EXPERTISE OF AN EXPERIENCED CRYSTALLOGRAPHIC REFEREE.

You have not supplied any structure factors. As a result the full set of tests cannot be run.

No syntax errors found. CIF dictionary
Please wait while processing .... Interpreting this report

Datablock: CTO

Bond precision: Co-Co = 0.0300 A Wavelength=0

Cell:	a=5.189(3)	b=5.189(3)	c=13.811(7)
	alpha=90	beta=90	gamma=120

Temperature: 300 K

	Calculated	Reported
Volume	322.1(5)	322.1(4)
Space group	R 3	R3
Hall group	R 3	?
Moiety formula	Co3 O6 Te	?
Sum formula	Co3 O6 Te	Co3 Te O6
Mr	400.39	400.39
Dx,g cm-3	6.193	0.000
Z	3	3
Mu (mm-1)	0.000	0.000
F000	143.9	0.0
F000'	542.89	
h,k,lmax		
Nref		
Tmin,Tmax		
Tmin'		

Correction method= Not given

Data completeness= Theta(max)=

R(reflections)= wR2(reflections)=

S = Npar=

The following ALERTS were generated. Each ALERT has the format
test-name_ALERT_alert-type_alert-level.
Click on the hyperlinks for more details of the test.

Alert level A

ATOM007_ALERT_1_A _atom_site_aniso_label is missing
Unique label identifying the atom site.
PLAT029_ALERT_3_A _diffrn_measured_fraction_theta_full value Low . 0.000 Why?
PLAT183_ALERT_1_A Missing _cell_measurement_reflns_used Value .... Please Do !
PLAT184_ALERT_1_A Missing _cell_measurement_theta_min Value ...... Please Do !
PLAT185_ALERT_1_A Missing _cell_measurement_theta_max Value ...... Please Do !
PLAT880_ALERT_1_A N0 datum for _diffrn_reflns_number .... Please Do !
PLAT881_ALERT_1_A No Datum for _diffrn_reflns_av_R_equivalents ... Please Do !

Alert level B

The above Alerts are N/A for crystal structure refined from powder Time-of-flight Neutron diffraction

PLAT701_ALERT_1_B Bond Calc 2.228(10), Rep 2.255(10), Dev.. 2.70 Sigma
CO1 -O1 1.555 1.555 .... # 1 Check

Alert level C

PLAT041_ALERT_1_C Calc. and Reported SumFormula Strings Differ Please Check

Alert level G

PLAT004_ALERT_5_G Polymeric Structure Found with Maximum Dimension 3 Info
PLAT005_ALERT_5_G No Embedded Refinement Details Found in the CIF Please Do !
PLAT104_ALERT_1_G The Reported Crystal System is Inconsistent with R3 Check
PLAT808_ALERT_5_G No Parseable SHELXL Style Weighting Scheme Found Please Check
PLAT882_ALERT_1_G No Datum for _diffrn_reflns_av_unetI/netI ...... Please Do !
PLAT883_ALERT_1_G No Info/Value for _atom_sites_solution_primary . Please Do !

7 **ALERT level A** = Most likely a serious problem - resolve or explain
1 **ALERT level B** = A potentially serious problem, consider carefully
1 **ALERT level C** = Check. Ensure it is not caused by an omission or oversight
6 **ALERT level G** = General information/check it is not something unexpected

11 ALERT type 1 CIF construction/syntax error, inconsistent or missing data
0 ALERT type 2 Indicator that the structure model may be wrong or deficient
1 ALERT type 3 Indicator that the structure quality may be low
0 ALERT type 4 Improvement, methodology, query or suggestion
3 ALERT type 5 Informative message, check

It is advisable to attempt to resolve as many as possible of the alerts in all categories. Often the minor alerts point to easily fixed oversights, errors and omissions in your CIF or refinement strategy, so attention to these fine details can be worthwhile. In order to resolve some of the more serious problems it may be necessary to carry out additional measurements or structure refinements. However, the purpose of your study may justify the reported deviations and the more serious of these should normally be commented upon in the discussion or experimental section of a paper or in the "special_details" fields of the CIF. checkCIF was carefully designed to identify outliers and unusual parameters, but every test has its limitations and alerts that are not important in a particular case may appear. Conversely, the absence of alerts does not guarantee there are no aspects of the results needing attention. It is up to the individual to critically assess their own results and, if necessary, seek expert advice.

Publication of your CIF in IUCr journals

A basic structural check has been run on your CIF. These basic checks will be run on all CIFs submitted for publication in IUCr journals (*Acta Crystallographica*, *Journal of Applied Crystallography*, *Journal of Synchrotron Radiation*); however, if you intend to submit to *Acta Crystallographica Section C* or *E* or *IUCrData*, you should make sure that full publication checks are run on the final version of your CIF prior to submission.

Publication of your CIF in other journals

Please refer to the *Notes for Authors* of the relevant journal for any special instructions relating to CIF submission.

Validation response form

Please find below a validation response form (VRF) that can be filled in and pasted into your CIF.

```
# start Validation Reply Form
_vrf_ATOM007_CTO
;
PROBLEM: _atom_site_aniso_label is missing
RESPONSE: ...
;
_vrf_PLAT029_CTO
;
PROBLEM: _diffrn_measured_fraction_theta_full value Low .      0.000 Why?
RESPONSE: ...
;
_vrf_PLAT183_CTO
;
PROBLEM: Missing _cell_measurement_reflns_used Value ....      Please Do !
RESPONSE: ...
;
_vrf_PLAT184_CTO
;
PROBLEM: Missing _cell_measurement_theta_min Value ......      Please Do !
RESPONSE: ...
;
_vrf_PLAT185_CTO
;
PROBLEM: Missing _cell_measurement_theta_max Value ......      Please Do !
```

RESPONSE: ...
;
_vrf_PLAT880_CTO
;
PROBLEM: N0 datum for _diffrn_reflns_number .... Please Do !
RESPONSE: ...
;
_vrf_PLAT881_CTO
;
PROBLEM: No Datum for _diffrn_reflns_av_R_equivalents ... Please Do !
RESPONSE: ...
;
_vrf_PLAT701_CTO
;
PROBLEM: Bond Calc 2.228(10), Rep 2.255(10), Dev.. 2.70 Sigma
RESPONSE: ...
;
end Validation Reply Form

PLATON version of 22/12/2019; check.def file version of 13/12/2019

Datablock CTO - ellipsoid plot

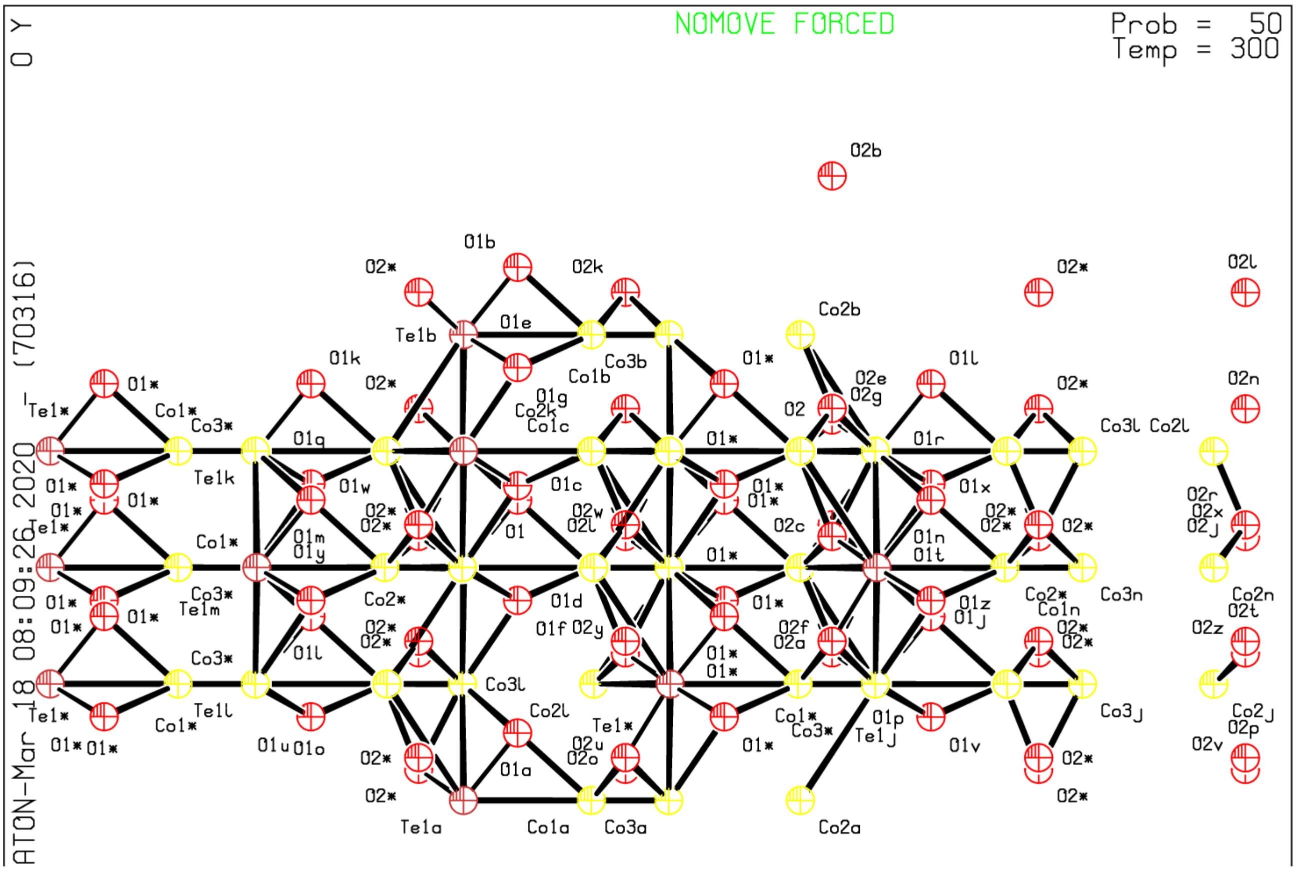

Download CIF editor (publCIF) from the IUCr
Download CIF editor (enCIFer) from the CCDC
Test a new CIF entry
